# A method of determining where to target surveillance efforts in heterogeneous epidemiological systems

**DOI:** 10.1371/journal.pcbi.1005712

**Published:** 2017-08-28

**Authors:** Alexander J. Mastin, Frank van den Bosch, Timothy R. Gottwald, Vasthi Alonso Chavez, Stephen R. Parnell

**Affiliations:** 1 Ecosystems and Environment Research Centre, School of Environment and Life Sciences, University of Salford, Greater Manchester, United Kingdom; 2 Computational and Systems Biology, Rothamsted Research, Harpenden, Hertfordshire, United Kingdom; 3 USDA Agricultural Research Service, Fort Pierce, Florida, United States of America; University of New South Wales, AUSTRALIA

## Abstract

The spread of pathogens into new environments poses a considerable threat to human, animal, and plant health, and by extension, human and animal wellbeing, ecosystem function, and agricultural productivity, worldwide. Early detection through effective surveillance is a key strategy to reduce the risk of their establishment. Whilst it is well established that statistical and economic considerations are of vital importance when planning surveillance efforts, it is also important to consider epidemiological characteristics of the pathogen in question—including heterogeneities within the epidemiological system itself. One of the most pronounced realisations of this heterogeneity is seen in the case of vector-borne pathogens, which spread between ‘hosts’ and ‘vectors’—with each group possessing distinct epidemiological characteristics. As a result, an important question when planning surveillance for emerging vector-borne pathogens is where to place sampling resources in order to detect the pathogen as early as possible. We answer this question by developing a statistical function which describes the probability distributions of the prevalences of infection at first detection in both hosts and vectors. We also show how this method can be adapted in order to maximise the probability of early detection of an emerging pathogen within imposed sample size and/or cost constraints, and demonstrate its application using two simple models of vector-borne citrus pathogens. Under the assumption of a linear cost function, we find that sampling costs are generally minimised when either hosts or vectors, but not both, are sampled.

## Author summary

Emerging pathogens are an increasing threat to human, animal, and plant health. In areas where these pathogens have not yet become established, surveillance is needed to detect incursions early enough to implement control measures. However, most epidemiological systems are heterogeneous in nature, and it is unclear how finite surveillance resources should be divided between constituent groups (such as hosts and vectors in the case of vector-borne pathogens). We use mathematical and statistical methods to address this issue. Taking the example of vector-borne pathogens, we show how to estimate the proportion of infected hosts or vectors at the time of first detection for any combination of host and vector sampling rates, given some knowledge of the characteristics of pathogen spread within and between hosts and vectors. We predict that the required total sampling effort and cost for early detection will be lowest when either hosts or vectors are sampled, with the optimal group to sample being the one with the highest estimated prevalence during initial exponential growth (which has clear parallels with ‘targeted surveillance’). We demonstrate the use of our framework by applying it to two vector-borne diseases of citrus and evaluate its predictions using a simple simulation model of sampling.

## Introduction

Human activities over the past 500 years have dramatically altered the distribution of organisms worldwide, through both purposeful and unintentional ‘invasions’ and ‘extinctions’ [1]. The global spread of plant pathogens, driven largely by the movement of people, plants, and products, as a result of globalisation [2–6], is an area of increasing concern, as these pathogens are a threat to natural ecosystems [7, 8] and horticultural industries [9–12] worldwide. The resilience of natural and managed ecosystems to new pathogens is further reduced by changes in land use and modern agricultural practices such as intensification, geographical consolidation, artificial selection, and genetic homogenisation [3, 13].

Plant disease control has historically been reactive in nature, but there is an increasing move towards proactive, risk-based, prevention strategies [14]. National and regional plant protection organisations therefore expend considerable effort in minimising the risk of emerging pathogens entering and establishing in new areas, through trade and movement restrictions/controls [15], border inspection and treatment [16], and ‘early detection surveillance’ activities [17]. Whilst movement restrictions and border checks help to minimise the risk of pathogen entry, early detection surveillance aims to detect pathogens following entry at a sufficiently early stage to allow control measures to be instigated. A failure of early detection may result in higher overall costs of control [16], or the loss of ability to control the pathogen altogether [18, 19]. It is well recognised that statistical and economic issues should be considered when planning early detection surveillance activities [20–23], but our previous work has shown that biological characteristics of the pathogen in question, in particular the rate of spread in a naive ecosystem, should also be considered [17, 24, 25]. This is particularly important in the case of emerging pathogens, where the prevalence of the invading epidemic will not be known until the time of first discovery, but can be approximated if the initial rate of transmission can be estimated.

Although many surveillance strategies are inherently founded on the assumption that the infection status of each individual is independent of all other individuals in the population (as is seen when simple random sampling is assumed to take place throughout the whole population), most epidemiological systems are characterised by marked heterogeneities [26, 27]. In theses cases, pathogens tend to spread within and between distinct ‘groups’ of individuals: such as between different hosts, to and from environmental reservoirs, and between hosts and disease-carrying vectors. Although these groupings, or ‘heterogeneities’, may be implicitly acknowledged during surveillance planning for logistical reasons, sampling strategies are commonly driven by ease of sampling, availability, or perceived importance. For example, surveillance strategies for plant diseases in particular have historically been largely based upon visual inspection of plants for signs of disease (despite this strategy delaying the timing of early detection [28, 29], reducing the ability to predict major disease outbreaks [30], and reducing the accuracy of prevalence estimation [12, 31]). Despite this, there has been increasing recognition in recent years of the potential to capitalise on heterogeneities in epidemiological systems by explicitly targeting early detection surveillance activities towards those groups which have a higher probability of infection (termed ‘risk-based’, or ‘targeted’, surveillance [26]) [32–34]. Although quantitative methods for targeting surveillance resources according to the risk of infection in a spatial context [35], or according to other epidemiological groupings [26] are available, these are often based on a largely ‘phenomenological’ interpretation of ‘risk groups’ (such as those obtained from statistical models). Few studies to date have attempted to develop a generic, biologically informed, framework for allocation of surveillance resources in heterogeneous systems based on a ‘mechanistic’ model of pathogen spread.

We focus here on vector-borne plant pathogens. These are responsible for a number of diseases of current concern, including huanglongbing (caused by bacteria of the genus *Candidatus* Liberibacter, and spread by hemipteran psyllid insects); olive quick decline syndrome (caused by the bacteria *Xylella fastidiosa* and spread predominantly by the Meadow spittlebug, *Philaenus spumarius*); and citrus tristeza syndromes (caused by the citrus tristeza virus and spread most effectively by the brown citrus aphid, *Toxoptera citricida* Kirkaldy). In the case of these pathogens, the aforementioned general focus on visual inspection for diagnosis means that commercially important host crops rather than insect vectors are often the primary focus of surveillance, as this is where symptoms and economic impacts are manifested, despite the recognised benefits of laboratory-based vector surveillance for diseases with long asymptomatic periods [28, 29].

In the current paper, we show how a mechanistic mathematical model of pathogen transmission can be linked with a statistical model of the timing of first detection during an ongoing surveillance campaign in order to estimate the mean prevalence at first detection in either group. As well as the total ‘sampling effort’ (the ‘rate’ of sample collection per unit time) from each group, the prevalence at first detection is affected by epidemiological characteristics of the pathogen in question (the rate of epidemic growth in the system as a whole and the relative prevalences of infection in hosts and vectors as the pathogen spreads through the system). We go on to show that the total sampling effort is minimised when either vectors or hosts, but not both, are sampled, and how the ‘costs’ of sampling can be incorporated into this framework.

## Methods

### Summary

The central question we wish to answer is what prevalence will a pathogen reach in hosts and in vectors when it is first detected (‘detection-prevalence’), given we know the sample size and the frequency of sampling from these two groups. In order to answer this, we develop (i) a statistically-based sampling model; and (ii) a mathematical model of pathogen population dynamics, which we combine in order to generate a heuristic (rule of thumb) for estimation of the prevalence at first detection and other useful outputs. Finally, we validate our heuristic by comparing its predictions with those obtained from a simulation model of host or vector sampling. In this section, we first outline the statistical sampling model, then go on to demonstrate how this can be parameterised using a compartmental mathematical model of the pathosystem in question, and finish by describing the two mathematical models we developed for this study.

### Statistical sampling model

We start by describing how a simple binomial-based sampling model can be used to estimate the prevalence at first detection in a heterogeneous system comprised of ‘hosts’ and ‘vectors’. The reader is referred to our earlier reports [17, 24, 25] and S1 Text for additional information on the derivation described below.

If we know the prevalence of infection at any specified timepoint, *t*_1_, we can use the binomial distribution to calculate the probability of failing to sample an infected individual during a single sampling round at time *t*_1_:

$$\begin{array}{c} {\left(1-\left[\frac{I\left(t_{1}\right)}{\rho }\right]\right)}^{N} \end{array}$$ (1)

Where *N* is the number of samples collected, and the ratio of the number of infected individuals at time *t*_1_, *I*(*t*_1_), to the total number of individuals, *ρ*, is the prevalence of infection. We define ‘prevalence’ as the proportion of infected individuals, which is commonly referred to as the ‘incidence’ in the field of plant pathology. From this, we can estimate the probability of at least one detection during sampling at this time (*P*(*t*_1_)):

$$\begin{array}{c} P\left(t_{1}\right)=1-{\left(1-\left[\frac{I\left(t_{1}\right)}{\rho }\right]\right)}^{N} \end{array}$$ (2)

The probability of first detection at time *t*_1_ in an ongoing sampling programme (where *N* samples are collected every Δ days) can be estimated as the product of eq 1 for each each of the *K* sampling points since initial entry of the pathogen (at time *t*_0_), and Eq 2:

$$\begin{array}{c} P\left(t_{1}|t_{0}\right)=\left[1-{\left(1-\left[\frac{I\left(t_{1}\right)}{\rho }\right]\right)}^{N}\right]\cdot \prod_{k=1}^{K}\left(1-{\left[\frac{I\left(t_{1}-k\Delta \right)}{\rho }\right]}^{N}\right) \end{array}$$ (3)

We can expand the framework in eq 3 in order to incorporate two groups of interest. Assuming a host-vector system where the number of infected hosts is *I*_*h*_ and the number of infected vectors is *I*_*v*_, and the total numbers of hosts and vectors are given as *ρ*_*h*_ and *ρ*_*v*_, we obtain the following:

$$\begin{array}{ll} P\left(t_{1}|t_{0}\right)= & \left[1-\left({\left(1-\left[\frac{I_{h}\left(t_{1}\right)}{\rho_{h}}\right]\right)}^{N_{h}}\cdot {\left(1-\left[\frac{I_{v}\left(t_{1}\right)}{\rho_{v}}\right]\right)}^{N_{v}}\right)\right]\\ & \cdot \prod_{k=1}^{K}\left({\left(1-\left[\frac{I_{h}\left(t_{1}-k\Delta \right)}{\rho_{h}}\right]\right)}^{N_{h}}\cdot {\left(1-\left[\frac{I_{v}\left(t_{1}-k\Delta \right)}{\rho_{v}}\right]\right)}^{N_{v}}\right) \end{array}$$ (4)

Where *N*_*h*_ is the number of hosts sampled at each sampling point, and *N*_*v*_ is the number of vectors sampled at each sampling point.

In reality, *t*_0_ is not known, but it is possible to estimate *t*_0_ given that detection occurs [17, 24]. First, we can simplify eq 4 if we assume that the initial increase in the prevalence is exponential in nature, that prevalences are low, and that sampling occurs as a continuous process rather than at discrete intervals (with a sampling rate of $\frac{N}{\Delta }=\theta$). Given that there is pathogen transmission between hosts and vectors in both directions, we can assume a single rate of exponential growth, *r*, for the system as a whole. We can then use Bayes’ theorem to represent the probability of first entry at time *t*_0_ given the pathogen was detected at time *t*_1_ [17, 24]:

$$\begin{array}{c} P(t_{0}|t_{1})\approx [(\theta_{h}[\frac{\nu_{h}}{\rho_{h}}]+\theta_{v}[\frac{\nu_{v}}{\rho_{v}}])e^{r(t_{1}-t_{0})}]\\ \;\cdot \text{exp}(-(\frac{1}{r})[(\theta_{h}[\frac{\nu_{h}}{\rho_{h}}]+\theta_{v}[\frac{\nu_{v}}{\rho_{v}}])e^{r(t_{1}-t_{0})}]) \end{array}$$ (5)

The two new parameters *ν*_*h*_ and *ν*_*v*_ can be interpreted as the relative numbers of infected hosts and vectors as exponential growth within the system as a whole is first achieved. Given that the initial increase in the number of infected hosts and vectors is exponential in nature, these estimates can be obtained from analysis of a system of ordinary differential equations (ODEs) (see the Sampling model parameterisation section below and S2 Text). If we assume deterministic growth in the prevalence over time, we can adjust eq 5 in order to calculate the expected prevalence, *q*, in each group at the time of first detection. Using the approach described in our previous work [17, 24] and in S1 Text, we find that the prevalence at first detection in each group (*κ* = *h*;*v*) follows an exponential distribution:

$$\begin{array}{c} P(q_{\kappa }^{*}|t_{1})\approx \lambda_{\kappa }e^{-\lambda_{\kappa }q_{\kappa }^{*}} \end{array}$$ (6)

The exponential rate parameter λ_*κ*_ in eq 6 will vary depending upon whether the prevalence in hosts or vectors is desired. For the host prevalence at first detection, λ_*h*_ is used, and is calculated as:

$$\begin{array}{c} \lambda_{h}=\left(\left(\frac{1}{r}\right)\left(\theta_{h}+\theta_{v}\left[\frac{\left(\frac{\nu_{v}}{\rho_{v}}\right)}{\left(\frac{\nu_{h}}{\rho_{h}}\right)}\right]\right)\right) \end{array}$$ (7)

For the vector prevalence at first detection, λ_*v*_ is used, and is calculated as:

$$\begin{array}{c} \lambda_{v}=\left(\left(\frac{1}{r}\right)\left(\theta_{h}\left[\frac{\left(\frac{\nu_{h}}{\rho_{h}}\right)}{\left(\frac{\nu_{v}}{\rho_{v}}\right)}\right]+\theta_{v}\right)\right) \end{array}$$ (8)

The values of *r*, $\left(\frac{\nu_{h}}{\rho_{h}}\right)$, and $\left(\frac{\nu_{v}}{\rho_{v}}\right)$ are estimated from a mathematical model of the pathosystem under study as described in the Sampling model parameterisation section below.

### Estimating sampling effort

The mean prevalences at first detection ($E(q_{h}^{*})$ and $E(q_{v}^{*})$) can be estimated as the inverse of the rate (λ_*κ*_) parameters of the exponential distributions in eqs 6 to 8:

$$\begin{array}{c} E\left(q_{h}^{*}\right)=\frac{r}{\left(\theta_{h}+\theta_{v}\left[\frac{\left(\frac{\nu_{v}}{\rho_{v}}\right)}{\left(\frac{\nu_{h}}{\rho_{h}}\right)}\right]\right)} \end{array}$$ (9)

$$\begin{array}{c} E\left(q_{v}^{*}\right)=\frac{r}{\left(\theta_{h}\left[\frac{\left(\frac{\nu_{h}}{\rho_{h}}\right)}{\left(\frac{\nu_{v}}{\rho_{v}}\right)}\right]+\theta_{v}\right)} \end{array}$$ (10)

When there is only one group of interest (i.e. only one group is sampled, and the mean prevalence in that group is to be estimated), these formulae reduce down to our original rule of thumb [17, 24]:

$$\begin{array}{c} E(q^{*})=\frac{r}{\theta }=\frac{r\Delta }{N} \end{array}$$ (11)

Eqs 9 and 10 can also be rearranged in order to estimate the rate of sampling required from each group in order to first detect the pathogen at a specified mean prevalence in either group, which we define here as the ‘sampling effort’. This gives four separate linear equations which represent the sampling effort required for first detection in hosts or vectors at any specified mean prevalence as a function of *r*, the ratio $\left[\frac{\left(\frac{\nu_{h}}{\rho_{h}}\right)}{\left(\frac{\nu_{v}}{\rho_{v}}\right)}\right]$, and the sampling effort from the other group (shown in S1 Text). Interestingly, we found that these are linear functions, meaning that the sampling rate will not be minimised by sampling from both groups and indicating that a single group alone should be sampled in order to minimise the total sampling effort. Representing the sampling effort when only one group is sampled as *Θ*, we can manipulate eqs 9 and 10 to show how to calculate the relative required rate of exclusive vector sampling, *Θ*_*v*_ (as compared to exclusive host sampling, *Θ*_*h*_) for detection at any specified mean prevalence (more details on this derivation can be found in S1 Text):

$$\begin{array}{c} \left(\frac{\Theta_{v}}{\Theta_{h}}\right)=\left[\frac{\left(\frac{\nu_{h}}{\rho_{h}}\right)}{\left(\frac{\nu_{v}}{\rho_{v}}\right)}\right] \end{array}$$ (12)

### Estimating sampling costs

In many cases, the main constraint to planned surveillance activities will be the resources available for sampling. If we assume that sampling will be conducted from either hosts or vectors, and that the total ‘cost’ of sampling from either group during each sampling round (which may be purely financial cost, or some other metric), *Z*_*h*_ or *Z*_*v*_, can be calculated as the sum of the ‘fixed’ costs of sampling from the group in question per sampling round (*ζ*_0*h*_ and *ζ*_0*v*_) and the product of the sampling effort and the cost of sampling a single individual from the group (*ζ*_*h*_ or *ζ*_*v*_):

$$\begin{array}{c} Z_{h}=\zeta_{0h}+\zeta_{h}\Theta_{h} \end{array}$$ (13)

$$\begin{array}{c} Z_{v}=\zeta_{0v}+\zeta_{v}\Theta_{v} \end{array}$$ (14)

This allows us to reformulate eq 12 as:

$$\left[\frac{\left(\frac{Z_{v}-\zeta_{0v}}{\zeta_{v}}\right)}{\left(\frac{Z_{h}-\zeta_{0h}}{\zeta_{h}}\right)}\right]=\left[\frac{\left(\frac{\nu_{h}}{\rho_{h}}\right)}{\left(\frac{\nu_{v}}{\rho_{v}}\right)}\right]$$ (15)

If we now assume that the total cost is constant and therefore equal regardless of which group is sampled (*Z*_*h*_ = *Z*_*v*_), and that the fixed costs of surveillance are also equal for either group (*ζ*_0*h*_ = *ζ*_0*v*_), then the left side of eq 15 reduces down to the ratio $\left(\frac{\zeta_{h}}{\zeta_{v}}\right)$. Under these constraints, the ratio $\left[\frac{\left(\frac{\nu_{h}}{\rho_{h}}\right)}{\left(\frac{\nu_{v}}{\rho_{v}}\right)}\right]$ therefore indicates the ratio of individual unit sampling costs at which the total cost of sampling exclusively from hosts would be equal to that when sampling exclusively from vectors. This can be also treated as a ‘threshold quantity’ which indicates whether to sample from hosts or vectors in order to minimise the total sampling cost:

- If $\left[\frac{\left(\frac{\nu_{h}}{\rho_{h}}\right)}{\left(\frac{\nu_{v}}{\rho_{v}}\right)}\right]>\left[\frac{\zeta_{h}}{\zeta_{v}}\right]$ then sample from hosts only.
- If $\left[\frac{\left(\frac{\nu_{h}}{\rho_{h}}\right)}{\left(\frac{\nu_{v}}{\rho_{v}}\right)}\right]=\left[\frac{\zeta_{h}}{\zeta_{v}}\right]$ then sample from hosts and/or vectors.
- If $\left[\frac{\left(\frac{\nu_{h}}{\rho_{h}}\right)}{\left(\frac{\nu_{v}}{\rho_{v}}\right)}\right]<\left[\frac{\zeta_{h}}{\zeta_{v}}\right]$ then sample from vectors only.

### Sampling model parameterisation

The rate of exponential increase for both groups, *r*, and the ratio $\left[\frac{\left(\frac{\nu_{h}}{\rho_{h}}\right)}{\left(\frac{\nu_{v}}{\rho_{v}}\right)}\right]$ can be estimated using techniques from conventional model stability analysis. If we create an ODE model of our epidemiological system and represent the number of infected individuals in the form of a matrix equation, we can extract the Jacobian matrix (the 2x2 matrix of partial differential equations describing the change in the number of infected individuals in each group):

$$\begin{array}{c} \left(\begin{array}{c} \dot{I_{h}}\\ \dot{I_{v}} \end{array})=(\begin{array}{cc} a & b\\ c & d \end{array})(\begin{array}{c} I_{h}\\ I_{v} \end{array}\right) \end{array}$$ (16)

The left side of eq 16 represents the derivative of the infected categories (represented here using dot notation rather than the Leibniz notation used earlier, for ease of visualisation): $\dot{I_{h}}$ represents the rate of change in the number of infected hosts, and $\dot{I_{v}}$ represents the rate of change in the number of infected vectors. The first term on the right of eq 16 is the Jacobian matrix, and the second term describes the current state of the infected hosts (*I*_*h*_) and vectors (*I*_*v*_).

We describe in S2 Text how eq 16 can be solved in order to estimate the number (and the proportion) of infected individuals at any time point during exponential growth, and how this relates to the ratio $\left[\frac{\left(\frac{\nu_{v}}{\rho_{v}}\right)}{\left(\frac{\nu_{h}}{\rho_{h}}\right)}\right]$. We therefore need to calculate the eigenvector (*ν*) associated with the dominant eigenvalue. We can do this by first calculating the trace (*T*) of the Jacobian matrix in eq 16 as (*a* + *d*), and the determinant (*D*) of the matrix as (*ad* − *bc*), which we can use to calculate the eigenvalues of the system. When we linearise our system around the disease-free steady state, the largest eigenvalue will approximate the initial exponential growth rate (*r*) for the system as a whole (i.e. the rate of increase in the number of both infected hosts and vectors):

$$\begin{array}{c} r\approx (\frac{T}{2})+\sqrt{(\frac{T^{2}}{4})-D} \end{array}$$ (17)

The ratio of the values of the eigenvector associated with this eigenvalue will describe the relative numbers of infected hosts and vectors $\left(\frac{\nu_{h}}{\nu_{v}}\right)$ as exponential growth proceeds. Since *r* is fixed for the system as a whole, this ratio captures the heterogeneities between host and vector infection as the pathogen spreads through the system. Assuming that there is some transmission between the two groups, the ratio of eigenvectors can be calculated using the following formula:

$$\begin{array}{c} \left(\frac{\nu_{h}}{\nu_{v}})\approx (\frac{r-d}{c})=(\frac{b}{r-a}\right) \end{array}$$ (18)

Multiplying eq 18 with the ratio of vector to host numbers gives us an estimate of the ratio $\left[\frac{\left(\frac{\nu_{h}}{\rho_{h}}\right)}{\left(\frac{\nu_{v}}{\rho_{v}}\right)}\right]$ (which can be interpreted as an estimate of the relative proportions of infected hosts and vectors—the relative prevalences—as exponential growth proceeds):

$$\begin{array}{c} \left(\frac{\left(\frac{\nu_{h}}{\rho_{h}}\right)}{\left(\frac{\nu_{v}}{\rho_{v}}\right)}\right)\approx \left(\frac{r-d}{c}\right)\left(\frac{\rho_{v}}{\rho_{h}}\right)=\left(\frac{b}{r-a}\right)\left(\frac{\rho_{v}}{\rho_{h}}\right) \end{array}$$ (19)

### Epidemiological models

To demonstrate our approach, we used a simple SI-type compartmental host-vector model framework [36] (described in S2 Text) to simulate the epidemiological dynamics of two important citrus pathogens. We used Southern Gardens Citrus, a commercial citrus plantation in south Florida, as the conceptual setting for our model, and parameterised the models as shown in Tables 1 and 2.

**Table 1:** Parameter values used in the estimation of the transmission parameters (*β*) for the two models in the current study.

Par	Interpretation	HLB model	Tristeza model
*T*	Duration of each visit (h)	5	6
*ϕ*	Rate of host visits by vector	1	1
*b*_*v*_	Rate of acquisition by vectors	$\frac{0.8}{42d}=0.02$	$\frac{0.8}{1d}=0.8$
*b*_*h*_	Rate of host inoculation	0.05	0.2

$\left(\beta_{\kappa }=(\frac{\phi }{\rho_{h}})(1-exp(-b_{\kappa }T))\right)$, where *κ* refers to the receiving group. Most estimates are taken from [29, 37] for HLB and [38, 39] for tristeza. The duration of feeding per visit for the HLB model was taken from [40] and for the tristeza model was adjusted according to the total efficiency of CTV transmission described in [41]. All rates (*ϕ*, *b*_*v*_, *b*_*h*_) are per day.

**Table 2:** Parameter values for different models used in the current study.

Par	Interpretation	HLB model	Tristeza model
*ρ*_*h*_	Number of hosts	250,000	250,000
*β*_*hv*_	Host-vector transmission rate	2e−8	7e−7
*μ*_*h*_	Rate of infected host removal	$(\frac{1}{365\times 5})=5e-4$	$(\frac{1}{365\times 10})=3e-4$
*τ*_*h*_	Rate of host recovery	0.0	0.0
*π*_*h*_	Prob of graft transmission	0.0	0.0
*ρ*_*v*_	Number of vectors	3,924,040	802,426
*β*_*vh*_	Vector-host transmission rate	4e−8	2e−7
*μ*_*v*_	Rate of infected vector removal	$\frac{1}{82}$	$\frac{1}{28}$
*τ*_*v*_	Rate of vector recovery	0.0	$\frac{1}{2}$
*π*_*v*_	Prob of transovarial transmission	0.036	0.0

All rates (*β*, *μ*, *τ*) are per day.

The full system of ODEs for the model framework are given in S2 Text, but the ODEs describing the numbers of infected hosts and vectors are as follows:

$$\begin{array}{c} \frac{dI_{h}}{dt}=S_{h}I_{v}\beta_{vh}+\left(\pi_{h}-1\right)\mu_{h}I_{h}-\tau_{h}I_{h} \end{array}$$ (20)

$$\begin{array}{c} \frac{dI_{v}}{dt}=S_{v}I_{h}\beta_{hv}+\left(\pi_{v}-1\right)\mu_{v}I_{v}-\tau_{v}I_{v} \end{array}$$ (21)

Linearising around the disease-free steady state, the components of the Jacobian matrix in eq 16 for this system can be calculated:

$$\begin{array}{c} a=\frac{\partial \dot{I_{h}}}{\partial I_{h}}\approx \mu_{h}(\pi_{h}-1)-\tau_{h} \end{array}$$ (22)

$$\begin{array}{c} b=\frac{\partial \dot{I_{h}}}{\partial I_{v}}\approx \rho_{h}\beta_{vh} \end{array}$$ (23)

$$\begin{array}{c} c=\frac{\partial \dot{I_{v}}}{\partial I_{h}}\approx \rho_{v}\beta_{hv} \end{array}$$ (24)

$$\begin{array}{c} d=\frac{\partial \dot{I_{v}}}{\partial I_{v}}\approx \mu_{v}(\pi_{v}-1)-\tau_{v} \end{array}$$ (25)

We used this model framework to create models of two insect-vectored citrus diseases of economic importance to the global citrus industry: huanglongbing (HLB) and tristeza diseases. The epidemiological unit in each model was an individual sweet orange tree (*Citrus × sinensis*) host, or single insect vector (Asian citrus psyllid, *Diaphorina citri* Kuwayama, or brown citrus aphid, *Toxoptera citricida* (Kirkaldy), respectively). Parameter values and sources for the two models are shown in Tables 1 and 2. We assume that there is no differential immigration and emigration of infected vectors [42], that the total number of hosts and vectors does not change over time, and that there was no vertical transmission amongst hosts due to certification and testing of budwood source trees [43]. We estimated transmission parameters using the approach described by Jeger and others [42, 44], and selected a suitable number of vectors to achieve an overall *R*_0_ of 100 when the number of hosts is fixed at 250,000 (see S2 Text). Our decision to fix the *R*_0_ for each pathosystem at 100 and use this to calculate the relative densities of hosts and vectors was primarily intended to account for the lack of data on vector abundance, and to allow comparison of different pathogen types [45, 46].

Huanglongbing (also known as citrus greening) is a fatal disease of citrus and related plants caused by phloem-restricted gram negative Alphaproteobacteria of the genus *Candidatus* Liberibacter [47]. The most common species of Liberibacter worldwide is *Ca.* L. asiaticus (Las), which is the cause of ‘Asian citrus greening’, and is spread by the phloem-feeding Asian citrus psyllid. Las can be considered a ‘persistently transmitted, circulative pathogen’ [45, 46]. These pathogens enter the haemolymph of the vector and have the potential for transovarial transmission (although this is disputed in the particular case of Las [37, 48]).

Unlike many plant viruses, the citrus tristeza virus (CTV) complex comprises a number of strains which are responsible for a wide range of syndromes in citrus and their relatives [49–51]. Although strains were traditionally differentiated according to disease phenotype [49, 52, 53], this relationship remains unclear [50, 54], and we therefore focus on the spread of an undefined ‘novel’ CTV strain by the brown citrus aphid (considered the most efficient vector of CTV [55]). CTV is considered a ‘semipersistently transmitted, foregut-borne’ pathogen [45, 46], which does not spread systemically and therefore is characterised by rapid acquisition [49, 52, 56] and short persistence [39, 57].

### Sampling simulation

In order to assess how well our sampling models (eqs 6 to 8) performed, we created a model to simulate the sampling process using a Monte Carlo approach [58] with 1000 iterations. For each iteration, we used the output of the full ODE transmission model to indicate the spread of our pathogen through a susceptible population, and simulated a sampling process during the resultant epidemic by randomly selecting a series of timepoints from the model output, accounting for the probability of detection at each. We used the specified sampling interval to estimate the timing of first sampling and the interval between subsequent samples, and we calculated the prevalence in hosts and vectors at each of these points, along with the probability of detection given the sample size (using the binomial sampling strategy described in eq 2). In order to convert these probabilistic estimates into a dichotomous classification of whether the pathogen was successfully detected or not, we generated a pseudorandom number between 0 and 1 for each sampling point and classified detection as ‘successful’ if this number was less than or equal to our estimated detection probability. We then recorded the earliest time of first detection for the iteration in question, and identified the associated host and vector prevalences at this point. We assumed that a total of 800 samples were collected and tested per month (based upon data provided by the United States Sugar Corporation to the Citrus Greening Symposium in 2009, detailing laboratory testing instigated in Southern Gardens Citrus during 2006 and 2007 [59]).

For ease of interpretation, we conducted most analysis assuming a cost ratio at the ‘threshold’ of $\left[\frac{\left(\frac{\nu_{h}}{\rho_{h}}\right)}{\left(\frac{\nu_{v}}{\rho_{v}}\right)}\right]$ (indicating the cost ratio at which point the total costs and the prevalences at first detection would be expected to be equal regardless of which stratum was sampled).

### Sensitivity analysis

We first investigated the effect of varying sampling effort on the mean prevalence at first detection amongst hosts and vectors. Since vector sampling was not routinely performed in Southern Gardens, we assumed similar parameters to host sampling (i.e. 800 vectors per month, individually tested). We then investigated the prevalence at first detection when either hosts or vectors are exclusively sampled within fixed cost constraints, and conducted a brief sensitivity analysis of our model parameter estimates on the model outputs, focussing on transmission rates (*β*). Centering on a ‘threshold’ cost ratio (as described above), we adjusted the parameter values by a factor of ten in order to investigate the effect on the value of the ratio $\left[\frac{\left(\frac{\nu_{h}}{\rho_{h}}\right)}{\left(\frac{\nu_{v}}{\rho_{v}}\right)}\right]$ and the mean prevalence in each stratum at first detection.

Analyses were conducted using R (version 3.3.1) [79] and the Anaconda distribution (version 2.4.0; Continuum Analytics, https://continuum.io/) of the Python programming language (version 3.5.0; Python Software Foundation, https://www.python.org/). Full code is provided in S3, S4, S5 and S6 Text.

## Results

The number of vectors per host required to achieve an *R*_0_ of 100 was 16 for the HLB model, and 3 for the tristeza model (reflecting the higher transmission rates in this model). The transmission dynamics of the two models over a period of three years (including the exponential growth approximation upon which the rule of thumb is based) are shown in S1 and S2 Figs.

Using the stability analysis technique described above, the rate of exponential growth (*r*) for the HLB system was 0.02, and the ratio $\left[\frac{\left(\frac{\nu_{h}}{\rho_{h}}\right)}{\left(\frac{\nu_{v}}{\rho_{v}}\right)}\right]$ (see eq 15) was 8. Assuming that the ratio of sampling costs was equal to this would mean that sufficient resources would be available to sample either 800 hosts or 6,382 vectors per month. As expected, when either hosts or vectors alone were sampled at this rate, the mean time of first detection from the simulation model was 266 days for exclusive host sampling, and 267 days for exclusive vector sampling. The simulation model and the heuristic both predicted a mean host prevalence of 0.0007 and a mean vector prevalence of 0.0001 at first detection, regardless of which group was sampled.

**Fig 1:**
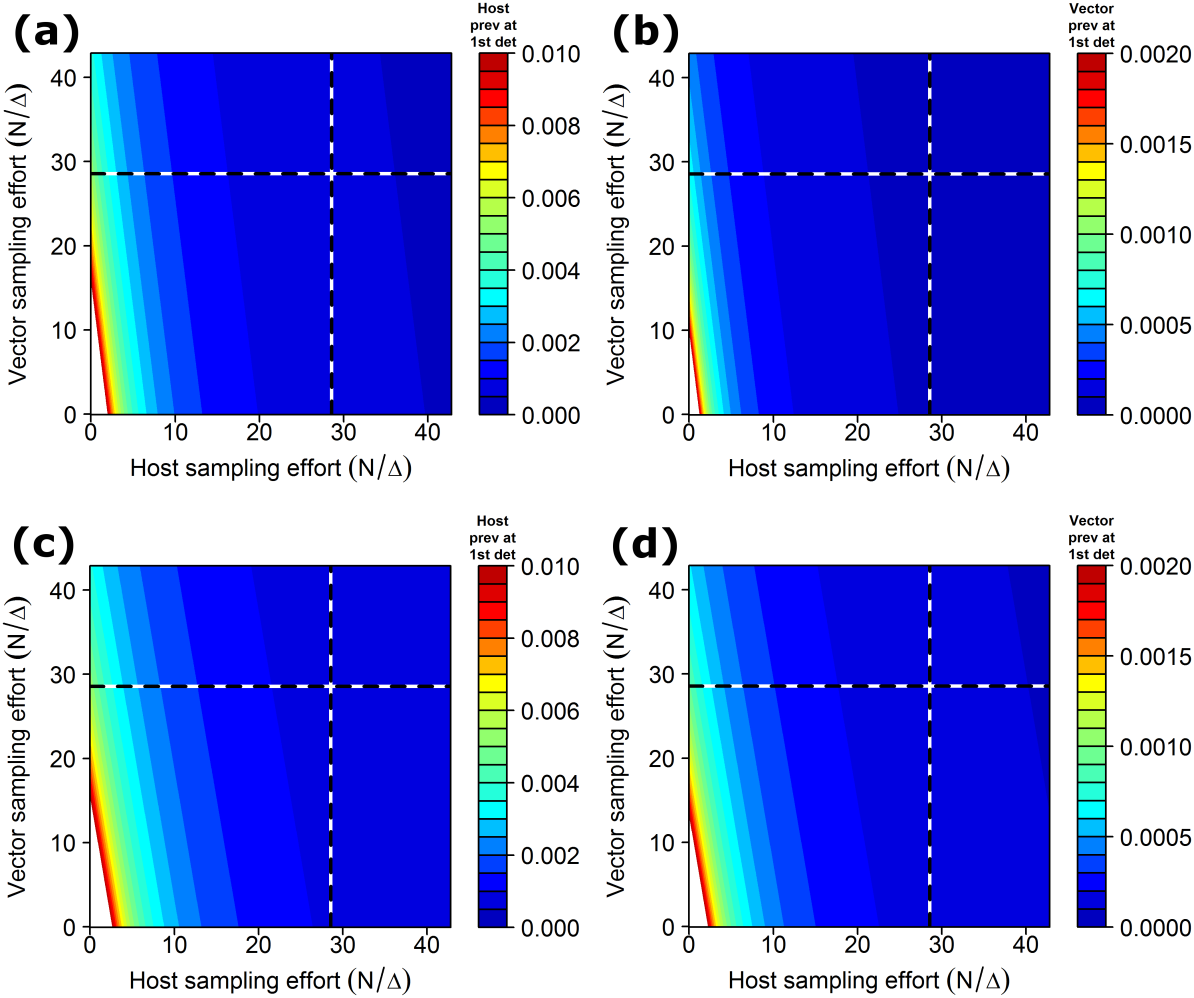
Effect of varying sampling effort $\left(\theta =\frac{N}{\Delta }\right)$ on the mean prevalence at first detection for the HLB model (panels (a) and (b) and the tristeza model (panels (c) and (d). The estimated prevalence at first detection in hosts is shown in the graphs on the left, and that in vectors is shown in the graphs on the right. The dashed line indicates a host (vertical line) and a vector (horizontal line) sampling effort of 800 samples per 28 days, with the intersection of these dashed lines indicating a theoretical scenario in which a total of 800 hosts and 800 vectors were sampled.

The estimate of the exponential growth parameter (*r*) obtained from the tristeza model was 0.03, and the ratio $\left[\frac{\left(\frac{\nu_{h}}{\rho_{h}}\right)}{\left(\frac{\nu_{v}}{\rho_{v}}\right)}\right]$ (eq 15) was 6. When the ratio of sampling costs was set to this, the available resources would allow sampling of either 800 hosts or 4,687 vectors every 28 days, which both gave a simulated time of first detection of 164 days. The heuristic predicted a mean prevalence in hosts at first detection of 0.0009 and the simulation model predicted a mean prevalence of 0.0010 at first detection, regardless of which group was sampled Similarly, amongst vectors, both methods predicted a mean prevalence of 0.0002, regardless of which group was sampled. Graphs of the distribution of the timing of first detection are shown in S3 and S4 Figs, and graphs of the prevalence distribution at first detection are shown in S3 and S4 Figs. Fig 1 shows the effect of varying the sampling effort (regardless of cost) on the mean prevalence at first detection using the heuristic described in eqs 9 and 10.

**Fig 2:**
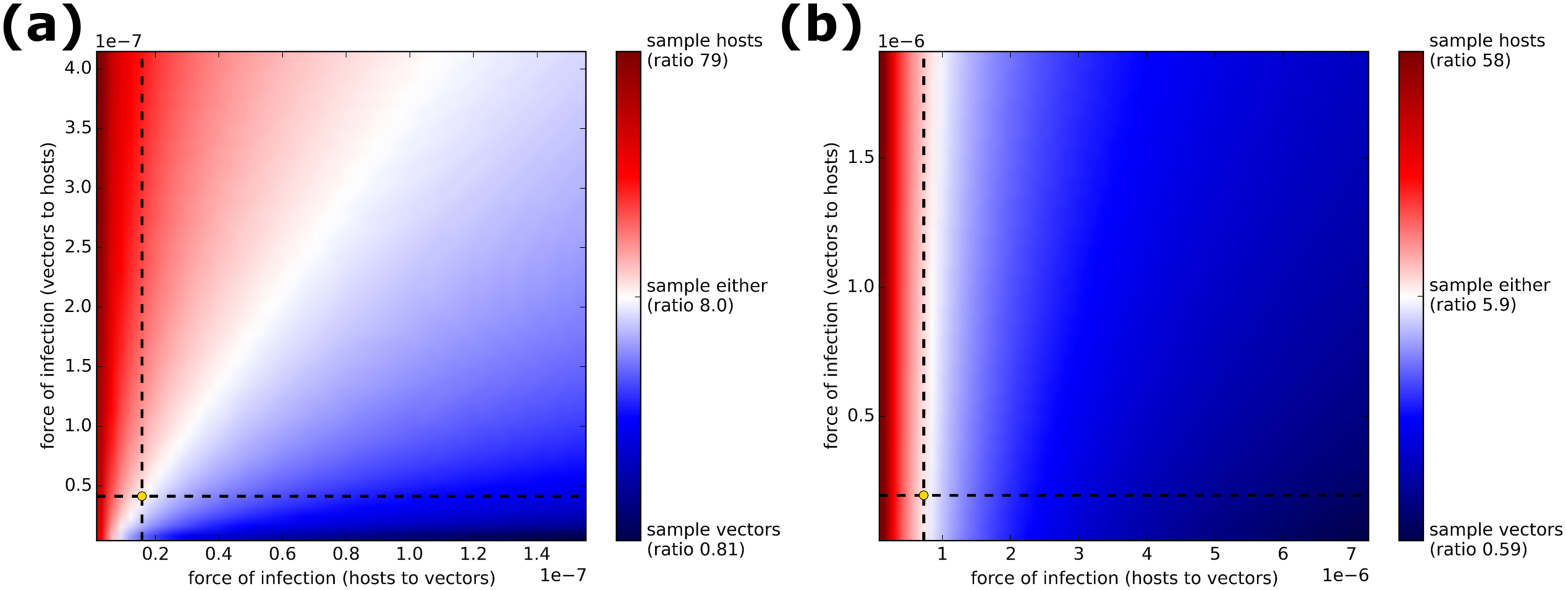
Effect of varying transmission parameters (*β*) on the suggested group of sampling for the HLB model (panel (a)) and the tristeza model (panel (b)). We estimate the relative sampling efforts required from vectors compared to that from hosts when using the current model parameters (located at the intersection of the dashed lines) using the ratio $\left[\frac{\left(\frac{\nu_{h}}{\rho_{h}}\right)}{\left(\frac{\nu_{v}}{\rho_{v}}\right)}\right]$, and assume that the relative cost of sampling hosts compared to vectors is equal to this threshold (8 for HLB, 6 for Tristeza)—indicating the ‘equivalence point’ as described in the text. The numbers in the key on the right describe the relative vector sampling effort $\left[\frac{\left(\frac{\nu_{h}}{\rho_{h}}\right)}{\left(\frac{\nu_{v}}{\rho_{v}}\right)}\right]$ for different transmission rates, but the colour gradient relates to the ratio of the relative vector sampling effort to the relative host sampling cost $\left[\frac{\zeta_{h}}{\zeta_{v}}\right]$, and is shown on the log scale in order to better discriminate values less than 1. Regions shown in red have a sampling effort ratio greater than the cost ratio (suggesting that sampling hosts would minimise the total cost) and those in blue have a ratio less than the cost ratio (suggesting that sampling vectors would minimise the total cost). The frontier between these two (indicating a ratio equal to the cost ratio) is shown in white.

Fig 2 shows the effect of varying the transmission parameters on the ratio $\left[\frac{\left(\frac{\nu_{h}}{\rho_{h}}\right)}{\left(\frac{\nu_{v}}{\rho_{v}}\right)}\right]$ (shown on the log scale to assist visualisation). Similar graphs for the host and vector longevity parameters, along with the effect of varying transmission and longevity parameters on the mean prevalence at first detection, are shown in S9–S12 Figs.

## Discussion

The protection of natural and managed ecosystems against the incursion of emerging pathogens increasingly relies upon the use of planned surveillance activities to detect pathogen entry at a suitably early time to allow control measures to be implemented. Failure of early detection can have catastrophic consequences, as was observed in the UK in 2001 following entry of the foot and mouth disease virus [60], and has been predicted due to chalara dieback of ash trees (caused by *Hymenoscyphus fraxineus*) throughout Europe [61, 62]. Although the risk of infection generally varies between different epidemiological groups within a single pathosystem, this heterogeneity has been commonly overlooked when planning surveillance activities. One particular example of this heterogeneity is seen in the case of pathogens spread by insect vectors, which are increasingly identified as ‘emerging pathogens’ and are a current source of considerable concern due to their potential impact upon animal and plant health [63]. Despite the clear epidemiological differences between ‘hosts’ and ‘vectors’, relatively little work has been conducted to date on how best to distribute surveillance resources between these groups in order to ensure that incursion of these emerging pathogens is rapidly detected by ongoing surveillance activities (‘early detection surveillance’).

In the current paper, we use vector-borne pathogens as an example of a ‘heterogeneous epidemiological system’, and describe how the prevalence at first detection in both hosts and vectors is related to the rate of sampling from these groups (eqs 6 to 8). We have developed a heuristic, which is parameterised using the rate of exponential growth, *r*, and a ratio, $\left[\frac{\left(\frac{\nu_{h}}{\rho_{h}}\right)}{\left(\frac{\nu_{v}}{\rho_{v}}\right)}\right]$, which describes the relative prevalences in each group during early exponential growth (and can also be interpreted as the relative sampling effort required from vectors when they are exclusively sampled, compared to that required from hosts). Both of these parameters can easily be obtained from a simple mathematical model of the system in question (as described in the Sampling model parameterisation section). Our heuristic is based upon an assumption of exponential growth in the prevalence of infection. Although this is unlikely to be epidemiologically realistic beyond the initial stages of epidemic growth, we find that our output (the probability of first detection), is constrained by the rapidly decreasing probability of having failed to detect the pathogen earlier, as reported in our previous work [17, 24]. Therefore, given a suitable sampling interval, the importance of being able to accurately estimate the prevalence decreases as the prevalence grows.

An important output of our method is the heuristic described in eqs 6 to 8. We can use this heuristic directly to evaluate ongoing or planned surveillance activities, in particular by predicting the distribution of prevalences (or mean prevalence) at first detection in either group, assuming a particular rate of sampling from each group. Alternatively, we can reformulate it in order to assist in surveillance planning, by estimating the sampling rate required in order to detect a specified mean prevalence (or specified prevalence percentile) in either group.

Another useful output of our work is shown in eq 15. This simple heuristic is focussed on direct interpretation of the ratio $\left[\frac{\left(\frac{\nu_{h}}{\rho_{h}}\right)}{\left(\frac{\nu_{v}}{\rho_{v}}\right)}\right]$, in order to determine whether hosts or vectors should be sampled in order to minimise both the prevalence at first detection and the total ‘cost’ of sampling, and can be used in two main ways:

- By explicitly specifying the sampling costs and adopting a dichotomised ‘threshold’ interpretation based upon the ratio $\left[\frac{\left(\frac{\nu_{h}}{\rho_{h}}\right)}{\left(\frac{\nu_{v}}{\rho_{v}}\right)}\right]$, such as that described above.
- By using the ratio $\left[\frac{\left(\frac{\nu_{h}}{\rho_{h}}\right)}{\left(\frac{\nu_{v}}{\rho_{v}}\right)}\right]$ to quantify the ratio of sampling costs at which the suggested sampling strategy would change (possibly in combination with sensitivity analysis, such as that shown in Fig 2). This strategy may be useful when the true ratio of sampling costs is less well known.

With the exception of our earlier work [17, 24, 25], the only other study we know of which attempted to develop a heuristic for evaluating early detection surveillance focussed on the estimation of the probability of detection before a specified prevalence was reached [64]. As our methods are able to estimate the whole probability distribution of the prevalence at the time of first detection, we are also able to estimate this probability if desired, along with measures such as the average prevalence at first detection.

The concept of surveillance within a host-vector system has been previously studied by Ferguson and others [65], who found that the relative prevalences and the costs of sampling determined the probability of detection in any group at any single sampling point. This shares similarities with our own formulation, since the ratio $\left[\frac{\left(\frac{\nu_{h}}{\rho_{h}}\right)}{\left(\frac{\nu_{v}}{\rho_{v}}\right)}\right]$ can be considered the ratio of prevalences amongst hosts and vectors during initial exponential growth. As with our approach, the Ferguson model had threshold-like behaviour in which the optimal sampling strategy suddenly changed, with the optimal sampling strategy generally being to focus on a single group of interest [65].

Finally, we considered similarities between our work and the body of literature on early detection surveillance within the more general field of invasion biology. Although a number of studies have investigated how to improve the early detection and control of invasive species, these generally considered the issue as an optimisation problem—using complex simulation models to determine the optimal strategy for surveillance and control, often in conjunction with economic modelling [22, 66–70]. Our method differs from these in that it does not require the creation of a complex model, but is still able to account for important biological properties of the pathosystem in question, including epidemiological groupings. Indeed, it has been argued that simple heuristics such as ours can be particularly useful for decision makers, since they can reduce a complex system down into a more manageable and understandable form [71].

Our framework assumes that the surveillance strategy in place is able to detect asymptomatic infection, and that the diagnostic test used is applied regardless of perceived infection status. As mentioned earlier, visual detection is the most commonly used method of first line diagnosis of infection status for plant pathogens, which is likely to be a problem for effective early detection surveillance [29], and is not compatible with our methodology in the case of most plant pathogens. Another repercussion of basing early detection surveillance strategies on visual detection is that consideration is rarely given to first line detection of infection in insect vectors (which generally do not show clinical signs following infection). Whilst we therefore suggest the use of highly sensitive tests able to detect asymptomatic infection, the additional immediate costs of applying these diagnostic methods means that extra consideration must be given to targeting those individuals most likely to be infected [30]. We achieve this in our framework by considering how to minimise total cost or effort by sampling exclusively from either hosts or vectors. However, our framework is equally capable of evaluating surveillance activities in which a combination of hosts and vectors are sampled, and could therefore be used by growers or regulatory agencies to help plan and evaluate ongoing surveillance activities.

We demonstrate the application of the current framework by developing two simple models of important vector-borne citrus pathogens: Las (the cause of HLB, which is a current emerging threat to the Californian citrus industry [72]) and CTV (the cause of citrus tristeza syndromes which have historically shaped the global citrus industry [49]) and base these models on a large plantation in south Florida. Despite arbitrarily setting *R*_0_ at 100, our estimates of *r* are comparable to those reported in the literature (*r* for Las has been estimated as between 0.002 and 0.01 [24], and that for CTV around 0.008 [73]). Our analysis of both the HLB and tristeza pathosystems suggested that sampling exclusively from hosts would minimise the total sampling effort, but that if the cost of sampling an individual host is more than eight times (sampling for Las) or six times (sampling for CTV) that of a single vector, vectors should instead be sampled in order to minimise total sampling costs. We do not attempt to estimate sampling costs, since it could be argued that pooled testing of multiple vectors together would raise the ratio of host to vector sample testing costs, whereas the additional effort required to capture motile vectors compared to sampling sessile hosts would lower the ratio of host to vector sample collection costs. Instead, we identify the ratio of sampling costs at which the suggested sampling strategy would change, using the ratio $\left[\frac{\left(\frac{\nu_{h}}{\rho_{h}}\right)}{\left(\frac{\nu_{v}}{\rho_{v}}\right)}\right]$ as described in eq 15 and the associated text. As well as correctly identifying this ‘equivalence point’ at which either hosts or vectors could reasonably be sampled in order to minimise sampling costs, our heuristic agreed well with results obtained from a simulated sampling model.

We investigated the effect of varying the transmission parameters on the suggested stratum of sampling. Fig 2 shows that an increase in host to vector transmission favoured vector sampling for both pathogens (associated with an increase in the relative prevalence amongst vectors), but that varying the rate of vector to host transmission only affected the suggested group of sampling in the HLB model (with higher values favouring host sampling). The lack of an effect of vector to host transmission in the tristeza model likely represents the constraining effect of the short duration of virus persistence in vectors.

Both the Las and CTV pathosystems are characterised by latent and incubation periods [12, 29, 74], and irregular distribution of the pathogen within the host [75–77], meaning that current available tests are imperfect (although work is currently in place to improve these tests). These characteristics would be expected to impact upon the optimal sampling strategy but are not explicitly captured in our current model. The issue of latency is a particularly important one for emerging plant pathogens [30], and has previously been used as an argument for sampling vectors instead of hosts for detection of Las [28]. It may be possible to adjust the statistical framework underlying our framework in order to capture latency [25] and imperfect test sensitivity, but incubation (where an individual is infected but not infectious) cannot be easily captured since the underlying mathematical model must be fully identifiable from the numbers of infected hosts and vectors at any time point. Also, we have purposefully selected a simple model for the costs of sampling and testing, with equal fixed costs and a linear cost function for variable costs. Further work is therefore needed to investigate the impact of these epidemiological and economic assumptions on model predictions, and to incorporate characteristics of importance into the framework.

Although we have described our approach using examples of host-vector systems, our framework should be applicable to any ‘heterogeneous’ epidemiological or ecological system—given that there is some transmission between the two groups (if this is not the case, each group should be sampled independently using our earlier frameworks [17, 24, 25]). As well as incorporating imperfect test performance and latency, further work will focus on investigation of the effect of nonlinear cost functions (since the per-sample collection cost would be expected to decrease as the surveillance intensity increases [78]), differences in fixed costs, generalisation to systems containing more than two linked epidemiological groups (offering the potential for investigating multiple hosts and/or vectors), ‘temporal targeting’ of surveillance effort by accounting for seasonality in the epidemiological system, and evaluation using more realistic, spatially explicit, transmission models.

### Conclusion

We propose an epidemiologically-informed approach to help answer the question of where best to place sampling resources for early detection of emerging pathogens in a system comprised of two epidemiologically distinct, but connected, groups (such as hosts and vectors). We show that the prevalence at first detection in each group can be estimated using a simple heuristic which, although novel, can be considered a generalisation of that from our own previous work [17, 24]. We demonstrate how to parameterise this heuristic using two epidemiological parameters which can be extracted from a system of ordinary differential equations: these are the initial rate of exponential growth of the pathogen in the system (*r*), and a ratio, $\left[\frac{\left(\frac{\nu_{h}}{\rho_{h}}\right)}{\left(\frac{\nu_{v}}{\rho_{v}}\right)}\right]$, which describes the relative prevalences in each group during exponential growth. We also show that the optimal strategy for minimising the total sample size (or the total sampling cost, if a linear cost function is assumed) will generally be to sample from a single group rather than both. Although this is contrary to many surveillance strategies, it is conceptually related to the idea of ‘risk-based’ surveillance, which is increasingly used for early detection surveillance. We have validated our approach using simple transmission models, but further work is needed to evaluate how well it performs in the face of more realistic, spatially explicit, transmission models.

## Supporting information

S1 Text Details on the full derivation of the sampling model. (PDF)

S2 Text Additional details on the transmission model. (PDF)

S3 Text Python 3.x code for sampling simulation model. (PY)

S4 Text Python 3.x code for single parameter sensitivity analysis model. (PY)

S5 Text Python 3.x code for dual parameter sensitivity analysis model. (PY)

S6 Text Simple R code for estimating the heuristic. (R)

S1 Fig HLB model transmission dynamics. Host and vector transmission dynamics in HLB model over the course of two years. Hosts are shown in panel (a) and vectors in panel (b). The relative densities of hosts and vectors for both models was fixed in order to give an *R*_0_ estimate of 100.
(TIF)

S2 Fig Tristeza model transmission dynamics. Host and vector transmission dynamics in the tristeza model over the course of two years. Hosts are shown in panel (a) and vectors in panel (b). The relative densities of hosts and vectors for both models was fixed in order to give an *R*_0_ estimate of 100.
(TIF)

S3 Fig Simulated distribution of time of first detection for the HLB model. Simulated distribution of time of first detection at the cost ratio threshold with a sampling ‘cost’ equivalent to that of 800 hosts every 28 days in the HLB model (i.e. either 800 hosts or 6,382 vectors). Panel (a) shows the results predicted when sampling 800 hosts and no vectors, and Panel (b) shows those predicted when sampling 6,382 vectors and no hosts. The dotted lines show the average time at first detection.
(TIF)

S4 Fig Simulated distribution of time of first detection for the tristeza model. Predicted distribution of time of first detection at the cost ratio threshold with a sampling ‘cost’ equivalent to that of 800 hosts every 28 days in the tristeza model (i.e. either 800 hosts or 4,687 vectors). Panel (a) shows the results predicted when sampling 800 hosts and no vectors, and Panel (b) shows those predicted when sampling 4,687 vectors and no hosts. The dotted lines show the average time at first detection.
(TIF)

S5 Fig Predicted distribution of prevalence at first detection for the HLB model. Predicted distribution of prevalence at first detection at the cost ratio threshold with a sampling ‘cost’ equivalent to that of 800 hosts every 28 days in the HLB model (i.e. either 800 hosts or 6,382 vectors), using both model simulation and the heuristic (‘rule of thumb’). Host prevalence at first detection is shown in panels (a) and (c), and vector prevalence in panels (b) and (d). Panels (a) and (b) show the results when sampling only from hosts, and panels (c) and (d) show those predicted when vectors alone are sampled. Dotted lines show the mean prevalence at first detection.
(TIF)

S6 Fig Predicted distribution of prevalence at first detection for the tristeza model. Predicted distribution of prevalence at first detection at the cost ratio threshold with a sampling ‘cost’ equivalent to that of 800 hosts every 28 days in the tristeza model (i.e. either 800 hosts or 4,687 vectors), using both model simulation and the heuristic (‘rule of thumb’). Host prevalence at first detection is shown in panels (a) and (c), and vector prevalence in panels (b) and (d). Panels (a) and (b) show the results when sampling only from hosts, and panels (c) and (d) show those predicted when vectors alone are sampled. Dotted lines show the mean prevalence at first detection.
(TIF)

S7 FigEffect of varying longevity parameters (*μ*) on the suggested group of sampling.Effect of varying longevity parameters (*μ*) on the suggested group of sampling for the HLB model (panel (a)) and the tristeza model (panel (b)), assuming a sampling cost ratio at the threshold (8 for HLB, 6 for Tristeza). The intersection of the dashed lines shows the current parameter values. The colour gradient relates to the ratio $\left[\frac{\left(\frac{\nu_{h}}{\rho_{h}}\right)}{\left(\frac{\nu_{v}}{\rho_{v}}\right)}\right]$, and is shown on the log scale. Red indicates a ratio greater than the cost ratio (suggesting host sampling) and blue indicates a ratio less than the cost ratio (suggesting vector sampling).(TIF)

S8 FigEffect of varying numbers of hosts and vectors (*ρ* parameters) on the suggested group of sampling.Effect of varying numbers of hosts and vectors (*ρ* parameters) on the suggested stratum of sampling for the HLB model (panel (a)) and the tristeza model (panel (b)), assuming a sampling cost ratio at the threshold (8 for HLB, 6 for Tristeza). The intersection of the dashed lines shows the current parameter values. The colour gradient relates to the ratio $\left[\frac{\left(\frac{\nu_{h}}{\rho_{h}}\right)}{\left(\frac{\nu_{v}}{\rho_{v}}\right)}\right]$, and is shown on the log scale. Red indicates a ratio greater than the cost ratio (suggesting host sampling) and blue indicates a ratio less than the cost ratio (suggesting vector sampling).(TIF)

S9 Fig Effect of varying transmission rates (*β* parameters) on the mean prevalence at first detection for the HLB model. Effect of varying transmission rates (*β* parameters) on the mean prevalence at first detection for the HLB model (host prevalence shown in panels (a) and (c) and vector prevalence in panels (b) and (d)). Red lines show the estimated prevalence when 800 hosts are sampled every 28 days, and blue lines show the estimated prevalence when 6,382 vectors are sampled every 28 days. Plots in panels (a) and (b) show the effect of varying host to vector transmission, and those in panels (c) and (d) show the effect of varying vector to host transmission. The dashed line shows the parameter value used in the model. The transmission parameters have units of ‘infections per host per vector per day’
(TIF)

S10 Fig Effect of varying transmission rates (*β* parameters) on the mean prevalence at first detection for the tristeza model. Effect of varying transmission rates (*β* parameters) on the mean prevalence at first detection for the tristeza model (host prevalence shown on the left and vector prevalence on the right). Red lines show the estimated prevalence when 800 hosts are sampled every 28 days, and blue lines show the estimated prevalence when 4,687 vectors are sampled every 28 days. Plots in panels (a) and (b) show the effect of varying host to vector transmission, and those in panels (c) and (d) show the effect of varying vector to host transmission. The dashed line shows the parameter value used in the model. The transmission parameters have units of ‘infections per host per vector per day’
(TIF)

S11 Fig Effect of varying longevity (*μ* parameters) on the mean prevalence at first detection for the HLB model. Effect of varying longevity (*μ* parameters) on the mean prevalence at first detection for the HLB model (host prevalence shown on the left and vector prevalence on the right). Red lines show the estimated prevalence when 800 hosts are sampled every 28 days, and blue lines show the estimated prevalence when 6,382 vectors are sampled every 28 days. Plots in panels (a) and (b) show the effect of varying host longevity, and those in panels (c) and (d) show the effect of varying vector longevity. The dashed line shows the parameter value used in the model.
(TIF)

S12 Fig Effect of varying longevity (*μ* parameters) on the mean prevalence at first detection for the tristeza model. Effect of varying longevity (*μ* parameters) on the mean prevalence at first detection for the tristeza model (host prevalence shown on the left and vector prevalence on the right). Red lines show the estimated prevalence when 800 hosts are sampled every 28 days, and blue lines show the estimated prevalence when 4,687 vectors are sampled every 28 days. Plots in panels (a) and (b) show the effect of varying host longevity, and those in panels (c) and (d) show the effect of varying vector longevity. The dashed line shows the parameter value used in the model.
(TIF)
