# Supplementary material for: A method of determining where to target surveillance efforts in heterogeneous epidemiological systems
Source: PLoS Comput Biol. 2017 Aug 28;13(8):e1005712. doi: 10.1371/journal.pcbi.1005712 (PMC5591013; doi:10.1371/journal.pcbi.1005712)
Supplement: S1 Text — (PDF) [file pcbi.1005712.s001.pdf]

S1 Text
A Method of Determining Where to Target Surveillance Efforts in Heterogeneous Epidemiological Systems: Supporting Information on the Statistical Model

Alexander J. Mastin[1], Frank van den Bosch[2], Timothy R. Gottwald[3], Vasthi Alonso Chavez[2], and Stephen R. Parnell[1]

[1]Ecosystems and Environment Research Centre, School of Environment and Life Sciences, University of Salford, Greater Manchester, M5 4WT, UK
[2]Computational and Systems Biology, Rothamsted Research, Harpenden, Hertfordshire, AL5 2JQ, UK
[3]USDA Agricultural Research Service, 2001 South Rock Road, Fort Pierce, Florida 34945, USA

Sampling model

We created a statistically informed sampling model based upon the prevalence of infection (proportion of infected individuals) in each group, a group-specific sample size (N_h from hosts and N_v from vectors), and a sampling interval of Δ days. As described in the main text, if we assume that first detection occurs at a specified time (t_1) and that the prevalence at this time (and all previous sampling points) is known, we can estimate to probability of first detection as the product of the probabilities of at least one detection at the sampling point in question, and the probability of no detection in all previous samplings:

$$P\left(t_1 \mid t_0\right) = \left[1 - \left(\left(1 - \left[\frac{I_h\left(t_1\right)}{\rho_h}\right]\right)^{N_h} \cdot \left(1 - \left[\frac{I_v\left(t_1\right)}{\rho_v}\right]\right)^{N_v}\right)\right] \cdot \prod_{k=1}^{K} \left(\left(1 - \left[\frac{I_h\left(t_1 - k\Delta\right)}{\rho_h}\right]\right)^{N_h} \cdot \left(1 - \left[\frac{I_v\left(t_1 - k\Delta\right)}{\rho_v}\right]\right)^{N_v}\right) \quad (1)$$

In order to simplify this equation further, we make three assumptions:

- The prevalences of infection are low (meaning that we can model this as a Poisson rather than a binomial process).

- Sampling is a continuous process rather than occurring at discrete intervals, with a sampling rate of $\frac{N}{\Delta}$ - represented below as θ. This assumption requires the sampling interval to not be too large [1].
- The growth in the prevalence in each group is exponential (as would be expected in the early stages of an epidemic).

If we assume that the prevalences of infection prior to first detection $\left[\frac{I_h(t_1)}{\rho_h}\right]$ and $\left[\frac{I_v(t_1)}{\rho_v}\right]$ are low (as would be the case in the early stages of an epidemic), we can model the probability detection at time t_1 as a Poisson (rather than a binomial) process by linearising equation 1 using the first terms of a Taylor series expansion:

$$
\begin{aligned}
P\left(t_1 \mid t_0\right) \approx & \left[1 - \exp\left(-\left(N_h\left[\frac{I_h\left(t_1\right)}{\rho_h}\right] + N_v\left[\frac{I_v\left(t_1\right)}{\rho_v}\right]\right)\right)\right] \\
& \cdot \prod_{k=1}^{K} \exp\left(-\left(N_h\left[\frac{I_h\left(t_1 - k\Delta\right)}{\rho_h}\right] + N_v\left[\frac{I_v\left(t_1 - k\Delta\right)}{\rho_v}\right]\right)\right)
\end{aligned} \tag{2}
$$

If we assume that sampling occurs a continuous process rather than occurring at discrete intervals, we can simplify equation 2 further to obtain the following equation:

$$
\begin{aligned}
P\left(t_1 \mid t_0\right) \approx & \left[1 - \exp\left(-\left(N_h\left[\frac{I_h\left(t_1\right)}{\rho_h}\right] + N_v\left[\frac{I_v\left(t_1\right)}{\rho_v}\right]\right)\right)\right] \\
& \cdot \exp\left(\int_{t_0}^{t_1} -\left(\frac{1}{\Delta}\right)\left(N_h\left[\frac{I_h\left(t\right)}{\rho_h}\right] + N_v\left[\frac{I_v\left(t\right)}{\rho_v}\right]\right) dt\right)
\end{aligned} \tag{3}
$$

And finally, if we assume that the prevalence in each group grows exponentially (and assuming that the prevalence is low), then equation 3 can be further simplified to give:

$$
\begin{aligned}
P\left(t_1 \mid t_0\right) \approx & \left[N_h\left[\frac{I_h\left(t_0\right)}{\rho_h}\right] e^{rt_1} + N_v\left[\frac{I_v\left(t_0\right)}{\rho_v}\right] e^{rt_1}\right] \\
& \cdot \exp\left(-\left(\frac{1}{r}\right)\left[\left(\frac{N_h}{\Delta}\right)\left[\frac{I_h\left(t_0\right)}{\rho_h}\right] e^{r(t_1 - t_0)} + \left(\frac{N_v}{\Delta}\right)\left[\frac{I_v\left(t_0\right)}{\rho_v}\right] e^{r(t_1 - t_0)}\right]\right)
\end{aligned} \tag{4}
$$

Equation 4 describes the probability of first detection at time t_1 given pathogen entry at time t_0. Since the time of pathogen entry will generally not be known, we can convert this in order to describe the probability of first entry at time t_0 given the pathogen was first detected at time t_1 using Bayes' theorem [1, 2], to convert this to the probability of first entry at time t_0 given the pathogen was detected at time t_1. Indicating the 'sampling rates', $\left(\frac{N_h}{\Delta}\right)$ and $\left(\frac{N_v}{\Delta}\right)$, as θ_h and θ_v, the following equation is obtained:

$$P(t_0 \mid t_1) \approx \left[\left(\theta_h \left[\frac{I_h(t_0)}{\rho_h}\right] + \theta_v \left[\frac{I_v(t_0)}{\rho_v}\right]\right) e^{rt_1}\right] \cdot \exp\left(-\left(\frac{1}{r}\right)\left[\left(\theta_h \left[\frac{I_h(t_0)}{\rho_h}\right] + \theta_v \left[\frac{I_v(t_0)}{\rho_v}\right]\right) e^{r(t_1-t_0)}\right]\right) \quad (5)$$

The ratios $\left(\frac{I_h(t_0)}{\rho_h}\right)$ and $\left(\frac{I_v(t_0)}{\rho_v}\right)$ can be interpreted in terms of the output of stability analysis of the Jacobian matrix of the system (as described in the main text and S2 Text) as $\left(c\left[\frac{\nu_h}{\rho_h}\right]\right)$ and $\left(c\left[\frac{\nu_v}{\rho_v}\right]\right)$, giving the following equation:

$$P(t_0 \mid t_1) \approx \left[\left(\theta_h \left[\frac{\nu_h}{\rho_h}\right] + \theta_v \left[\frac{\nu_v}{\rho_v}\right]\right) Ce^{rt_1}\right] \cdot \exp\left(-\left(\frac{1}{r}\right)\left[\left(\theta_h \left[\frac{\nu_h}{\rho_h}\right] + \theta_v \left[\frac{\nu_v}{\rho_v}\right]\right) Ce^{r(t_1-t_0)}\right]\right) \quad (6)$$

Here, ν_h and ν_v are eigenvectors, and C is a constant - both of which are associated with the dominant eigenvalue of the Jacobian matrix (see S2 Text).

Finally, we convert this estimate of the timing of first detection to an estimate of the prevalence at first detection. As this prevalence will vary between groups, this stage requires consideration of each group independently. In order to convert $P(t_0 \mid t_1)$ to the probability of the epidemic reaching prevalence q^* in the group of interest (κ), given detection at time t_1 ($P(q^*_\kappa \mid t_1)$), we apply the following transformation to each group:

$$P(q^*_\kappa \mid t_1) = \left(\frac{1}{r}\right)\left(\frac{1}{q^*_\kappa}\right)\left(\frac{1}{1-q^*_\kappa}\right) P(t_0 \mid t_1) \quad (7)$$

Again, if we assume that the prevalence grows exponentially ($q^* \approx q_0 e^{rt}$), and that the prevalence is low (meaning that $\left(\frac{1}{1-q^*_\kappa}\right) \approx 1$, since $\left(\frac{\nu_K}{\rho_K}\right) ce^{rt_1} = \left(\frac{I_K(t_0)}{\rho_K}\right) e^{rt_1} = q_K(t_1)$ the following two formulae are obtained:

$$P(q^*_h \mid t_1) = \left(\frac{1}{r}\right)\left(\theta_h + \theta_v \left[\frac{\left(\frac{\nu_v(t_0)}{\rho_v}\right)}{\left(\frac{\nu_h(t_0)}{\rho_h}\right)}\right]\right) \cdot \exp\left(-\left(\frac{1}{r}\right)\left(\theta_h + \theta_v \left[\frac{\left(\frac{\nu_v(t_0)}{\rho_v}\right)}{\left(\frac{\nu_h(t_0)}{\rho_h}\right)}\right]\right) q^*_h\right) \quad (8)$$

$$P(q^*_v \mid t_1) = \left(\frac{1}{r}\right)\left(\theta_h \left[\frac{\left(\frac{\nu_h(t_0)}{\rho_h}\right)}{\left(\frac{\nu_v(t_0)}{\rho_v}\right)}\right] + \theta_v\right) \cdot \exp\left(-\left(\frac{1}{r}\right)\left(\theta_h \left[\frac{\left(\frac{\nu_h(t_0)}{\rho_h}\right)}{\left(\frac{\nu_v(t_0)}{\rho_v}\right)}\right] + \theta_v\right) q^*_v\right) \quad (9)$$

As described in the main text, equations 8 and 9 represent exponential distributions:.

$$P\left(q_h^* \mid t_1\right) \sim \text{Exponential}\left(\left(\frac{1}{r}\right)\left(\theta_h + \theta_v\left[\frac{\left(\frac{\nu_v}{\rho_v}\right)}{\left(\frac{\nu_h}{\rho_h}\right)}\right]\right)\right) \tag{10}$$

$$P\left(q_v^* \mid t_1\right) \sim \text{Exponential}\left(\left(\frac{1}{r}\right)\left(\theta_h\left[\frac{\left(\frac{\nu_h}{\rho_h}\right)}{\left(\frac{\nu_v}{\rho_v}\right)}\right] + \theta_v\right)\right) \tag{11}$$

The mean prevalences at first detection ($E\left(q_h^*\right)$ and $E\left(q_v^*\right)$) can be estimated as the inverse of the exponential rate parameter, according to the characteristics of the exponential distributions in Equations 10 and 11:

$$E\left(q_h^*\right) = \frac{r}{\left(\theta_h + \theta_v\left[\frac{\left(\frac{\nu_v}{\rho_v}\right)}{\left(\frac{\nu_h}{\rho_h}\right)}\right]\right)} \tag{12}$$

$$E\left(q_v^*\right) = \frac{r}{\left(\theta_h\left[\frac{\left(\frac{\nu_v}{\rho_h}\right)}{\left(\frac{\nu_h}{\rho_v}\right)}\right] + \theta_v\right)} \tag{13}$$

We can rearrange these to give an estimate of the sampling rate required for detection at a specified mean prevalence in either hosts ($E\left(q_h^*\right)$) or vectors ($E\left(q_v^*\right)$). Since we are also interested in both the sampling rate from hosts and vectors, this gives us a total of four equations:

The sampling rate required from hosts for detection at a specified mean host prevalence:

$$\theta_h = \left(\frac{r}{E\left(q_h^*\right)}\right) - \left[\frac{\left(\frac{\nu_v}{\rho_v}\right)}{\left(\frac{\nu_h}{\rho_h}\right)}\right]\theta_v \tag{14}$$

The sampling rate required from vectors for detection at a specified mean host prevalence:

$$\theta_v = \left(\frac{r}{E\left(q_h^*\right)}\right)\left[\frac{\left(\frac{\nu_h}{\rho_h}\right)}{\left(\frac{\nu_v}{\rho_v}\right)}\right] - \left[\frac{\left(\frac{\nu_h}{\rho_h}\right)}{\left(\frac{\nu_v}{\rho_v}\right)}\right]\theta_h \tag{15}$$

The sampling rate required from hosts for detection at a specified mean vector prevalence:

$$\theta_h = \left(\frac{r}{E\left(q_v^*\right)}\right)\left[\frac{\left(\frac{\nu_v}{\rho_v}\right)}{\left(\frac{\nu_h}{\rho_h}\right)}\right] - \left[\frac{\left(\frac{\nu_v}{\rho_v}\right)}{\left(\frac{\nu_h}{\rho_h}\right)}\right]\theta_v \tag{16}$$

The sampling rate required from vectors for detection at a specified mean vector prevalence:

$$\theta_v = \left(\frac{r}{E\left(q_v^*\right)}\right) - \left[\frac{\left(\frac{\nu_h}{\rho_h}\right)}{\left(\frac{\nu_v}{\rho_v}\right)}\right]\theta_h \tag{17}$$

These are linear equations (containing an 'intercept' and a 'slope' term), indicating that when sampling to detect a specified mean prevalence (in either hosts or vectors), either hosts or vectors (but not both) should generally be sampled. If this is the case, the 'intercept' term alone in equations 14 - 17 will describe the required sampling rate (which we describe as Θ_h and Θ_v, to distinguish the single group sampling rate from the combined sampling rate, θ, in equations 14-17). We can reformulate these new equations in terms of the expected prevalence at first detection. Using equations 14 and 15, we can describe the mean host prevalence at first detection as a function of the host or the vector sampling rate:

$$E\left(q_h^*\right) = \left(\frac{r}{\Theta_h}\right) = \left(\frac{r}{\Theta_v}\right)\left[\frac{\left(\frac{\nu_h}{\rho_h}\right)}{\left(\frac{\nu_v}{\rho_v}\right)}\right] \tag{18}$$

And using equations 16 and 17, we can describe the mean vector prevalence at first detection as a function of the host or the vector sampling rate:

$$E\left(q_v^*\right) = \left(\frac{r}{\Theta_h}\right)\left[\frac{\left(\frac{\nu_v}{\rho_v}\right)}{\left(\frac{\nu_h}{\rho_h}\right)}\right] = \left(\frac{r}{\Theta_v}\right) \tag{19}$$

Both of these formulations simplified down into the following equation, which describes the relative sampling rate required when vectors are exclusively sampled and when hosts are exclusively sampled, for any specified mean prevalence at first detection in either hosts or vectors:

$$\left(\frac{\Theta_v}{\Theta_h}\right) = \left[\frac{\left(\frac{\nu_h}{\rho_h}\right)}{\left(\frac{\nu_v}{\rho_v}\right)}\right] \tag{20}$$

The right side of equation 20 can be interpreted as a 'threshold quantity'. A value of greater than one indicates that the sampling rate required when sampling only from vectors is greater than that required when sampling only from hosts; and a value of less than one indicates the converse. We describe in the main paper how costs can be incorporated into this framework.

References

[1] Parnell, S., T. R. Gottwald, W. R. Gilks, and F. van den Bosch. 2012. Estimating the Incidence of an Epidemic When It Is First Discovered and the Design of Early Detection Monitoring. Journal of Theoretical Biology 305 (July): 3036.

[2] Parnell, S., T. R. Gottwald, N. J. Cunniffe, V. Alonso Chavez, and F. van den Bosch. 2015. Early Detection Surveillance for an Emerging Plant Pathogen: A Rule of Thumb to Predict Prevalence at First Discovery. Proceedings. Biological Sciences / The Royal Society 282 (1814): 20151478.
