# Supplementary material for: A method of determining where to target surveillance efforts in heterogeneous epidemiological systems
Source: PLoS Comput Biol. 2017 Aug 28;13(8):e1005712. doi: 10.1371/journal.pcbi.1005712 (PMC5591013; doi:10.1371/journal.pcbi.1005712)
Supplement: S2 Text — (PDF) [file pcbi.1005712.s002.pdf]

S2 Text
A Method of Determining Where to Target Surveillance Efforts in Heterogeneous Epidemiological Systems: Supporting Information on the Epidemiological Model

Alexander J. Mastin[1], Frank van den Bosch[2], Timothy R. Gottwald[3], Vasthi Alonso Chavez[2], and Stephen R. Parnell[1]

[1]Ecosystems and Environment Research Centre, School of Environment and Life Sciences, University of Salford, Greater Manchester, M5 4WT, UK
[2]Computational and Systems Biology, Rothamsted Research, Harpenden, Hertfordshire, AL5 2JQ, UK
[3]USDA Agricultural Research Service, 2001 South Rock Road, Fort Pierce, Florida 34945, USA

Transmission model

The conceptual structure of the models are shown in figure 1 and equations 1-4. Model parameters are described in table 1. We use absolute numbers of infected (and uninfected) individuals in our current framework, since the area occupied by the vector population is less likely to be constant [2].

$$\frac{dS_h}{dt} = (1 - \pi_h)\mu_h I_h + \tau_h I_h - S_h I_v \beta_{vh} \tag{1}$$

$$\frac{dI_h}{dt} = S_h I_v \beta_{vh} + (\pi_h - 1)\mu_h I_h - \tau_h I_h \tag{2}$$

$$\frac{dS_v}{dt} = (1 - \pi_v)\mu_v I_v + \tau_v I_v - S_v I_h \beta_{hv} \tag{3}$$

$$\frac{dI_v}{dt} = S_v I_h \beta_{hv} + (\pi_v - 1)\mu_v I_v - \tau_v I_v \tag{4}$$

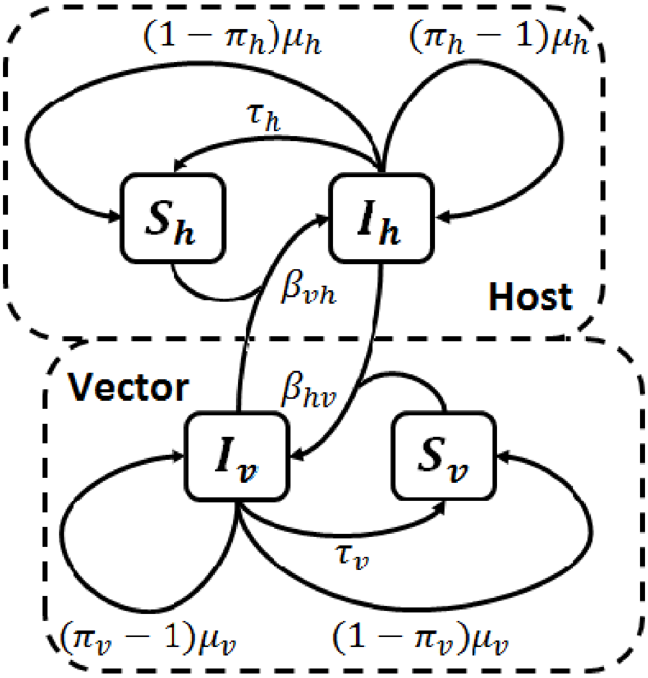

Figure 1: Structure of model used in current study

Derivation of the ratio $\left[\frac{\left(\frac{\nu_v}{\rho_v}\right)}{\left(\frac{\nu_h}{\rho_h}\right)}\right]$

As described in the main text, we can represent this simple epidemiological system in matrix form as follows:

$$\begin{pmatrix} \dot{I}_h \\ \dot{I}_v \end{pmatrix} = \begin{pmatrix} a & b \\ c & d \end{pmatrix} \begin{pmatrix} I_h \\ I_v \end{pmatrix} \tag{5}$$

Here, the term on the left side of the equation describes the derivative of the number of infected hosts and vectors; the first term on the right side of the equation is the Jacobian matrix, which describing the relative changes in the numbers of infected individuals in each group as the number in the other groups are changed; and the second term on the right describes the number of infected individuals in each group at the timepoint in question. If we assume exponential growth, this system can be solved analytically to give the following:

$$\begin{pmatrix} I_h(t) \\ I_v(t) \end{pmatrix} = C_1 \begin{pmatrix} \nu_{1h} \\ \nu_{1v} \end{pmatrix} e^{\lambda_1 t} + C_2 \begin{pmatrix} \nu_{2h} \\ \nu_{2v} \end{pmatrix} e^{\lambda_2 t} \tag{6}$$

Here, C_1 and C_2 are constants, λ_1 and λ_2 are eigenvalues, and ν_1 and ν_2 are the associated eigenvectors (each of length 2: individual entries are labelled here with the subscripts h and v). The largest eigenvalue (λ_1) will generally reflect the exponential growth rate (r) for the system as a whole, and the number of infected individuals in either group at any time point (t) can therefore be approximated as:

Par	Interpretation	Units
ρ_h	Number of hosts	(hosts)
β_{hv}	Transmission from hosts to vectors	Probability of acquisition of pathogen by vector given contact between infected host and susceptible vector (per host per vector per day)
μ_h	Rate of infected host turnover	Probability of host replacement (per day)
τ_h	Host recovery	Probability of infected host recovery (per day)
π_h	Vertical transmission of pathogen	Proportion of infected hosts which transmit pathogen to new individuals
ρ_h	Number of vectors	(vectors)
β_{vh}	Transmission from vectors to hosts	Probability of inoculation of host with pathogen given contact between infected vector and susceptible host (per host per vector per day)
μ_v	Rate of infected vector turnover	Probability of vector replacement (per day)
τ_v	Vector recovery	Probability of infected vector recovery (per day)
π_v	Transovarial transmission of pathogen	Proportion of infected female vectors which transmit pathogen to eggs/nymphs

Table 1: Description of model parameters

$$\begin{pmatrix} I_h(t) \\ I_v(t) \end{pmatrix} \approx C_1 \begin{pmatrix} \nu_{1h} \\ \nu_{1v} \end{pmatrix} e^{\lambda_1 t} \tag{7}$$

Taking each group separately, we can adjust this to describe the prevalence in each group of interest by dividing by the total population size ρ_i:

$$q_h(t) \approx C_1 \left(\frac{\nu_{1h}}{\rho_h} \right) e^{\lambda_1 t} \tag{8}$$

$$q_v(t) \approx C_1 \left(\frac{\nu_{1v}}{\rho_v} \right) e^{\lambda_1 t} \tag{9}$$

We can assess the relationship between the prevalence in vectors and the prevalence in hosts as exponential growth proceeds by taking the ratio of equa-

tions 9 and 8. Doing this, we obtain a central component of our framework: the ratio $\left[\frac{\left(\frac{\nu_v}{\rho_v}\right)}{\left(\frac{\nu_h}{\rho_h}\right)}\right]$:

$$\frac{q_v(t)}{q_h(t)} = \frac{C_1\left(\frac{\nu_{1v}}{\rho_v}\right)e^{\lambda_1 t}}{C_1\left(\frac{\nu_{1h}}{\rho_h}\right)e^{\lambda_1 t}} = \left[\frac{\left(\frac{\nu_v}{\rho_v}\right)}{\left(\frac{\nu_h}{\rho_h}\right)}\right] \tag{10}$$

Estimating transmission parameters and R0

A major challenge when developing dynamic models is characterising the association between the population density and the rate of transmission (β) between individuals. In order to describe this association, the terms 'frequency dependent' and 'density dependent' are commonly used [2] (although it is likely that these just represent two extremities of a spectrum of transmission patterns, with the 'true' situation in most cases lying somewhere inbetween [3, 6]). Vector-borne pathogens are commonly assumed to follow 'frequency-dependent' transmission, whereby the rate of contact (and therefore transmission) does not increase as the population size increases, since many vectors will actively seek out their hosts [7, 1]. In our model, we do not adopt either of the formulations described by Begon and others [2], but estimate the rate of transmission using the approach described by Jeger and others [4, 5], in which the rate of contact increases according to the relative numbers of vectors per host. This framework allows us to explicitly model the contact rate between hosts and vectors according to the total number of visits each vector makes to hosts per day (ϕ). When divided by the total number of hosts (ρ_h), this gives an estimate of the proportion of hosts visited by a single vector per day: $\left(\frac{\phi}{\rho_h}\right)$. The probability that this contact is infectious can be estimated as the product of the daily rate of inoculation (host infection, b_h) or acquisition (vector infection, b_v), and the duration of feeding per visit (in days) (T). As described by Madden and others [5], in order to prevent unreasonable estimates, this rate can be converted to a probability using the zeroth term of the Poisson: $(1-\exp(b_h T))$ for inoculation, or $(1-\exp(b_v T))$ for acquisition. The product of this estimate and the contact rate gives an estimate of the probability of infectious contact (given that one party is infectious and the other is susceptible), β:

$$\beta_{hv} = \left(\frac{\phi}{\rho_h}\right)(1-\exp(b_v T)) \tag{11}$$

$$\beta_{vh} = \left(\frac{\phi}{\rho_h}\right)(1-\exp(b_h T)) \tag{12}$$

Since these β parameters have units of 'infections per host per vector per day', we can estimate the number of vectors which acquire infection as the product of equation 11, the number of infected hosts, and the number of susceptible vectors. Similarly, the number of hosts which become inoculated can be estimated as the product of equation 12, the number of susceptible hosts, and the number of infected vectors. As β_{hv} and β_{vh} increase as the number of vectors per host increases, the relative numbers of hosts and vectors in the model has a considerable effect on the rate of transmission and therefore the model output.

Although the selection of the host population size (being constrained within a fixed area) can be achieved in a relatively arbitrary manner, the quantification of the total number of vectors associated with these plants is challenging due to overdispersion (vectors may be aggregated on individual hosts); seasonality (vector abundance is likely to vary seasonally); and a general lack of data (estimating absolute vector densities can be very challenging). As a result, we calculated the relative numbers of vectors per host according to a predetermined estimate of the basic reproduction number (R_0).

We can estimate R_0 for the system as follows:

$$R_0 = \left(\frac{\rho_h \beta_{vh}}{\tau_v + (\mu_v (1 - \pi_v))} \right) \left(\frac{\rho_v \beta_{hv}}{\tau_h + (\mu_h (1 - \pi_h))} \right) \tag{13}$$

Heuristically, this can be interpreted as the product of the total number of hosts infected by single vector over its infective lifespan (first term in parentheses), and the total number of vectors infected by a single host over its infective lifespan (second term in parentheses). We can reformulate this equation in order to estimate the required number of vectors (given that the number of hosts is known) for any specified R_0:

$$\rho_v = R_0 \left(\frac{(\tau_v + (\mu_v (1 - \pi_v))) (\tau_h + (\mu_h (1 - \pi_h)))}{\rho_h \beta_{vh} \beta_{hv}} \right) \tag{14}$$

References

[1] Antonovics, J., Iwasa, Y., and Hassell, M. P. 1995. "A generalized model of parasitoid venereal and vector based transmission processes." American Naturalist 145 (5): 661675.

[2] Begon, M., Bennett, M., Bowers, R. G., French, N. P., Hazel, S. M., and Turner, J. 2002. "A clarification of transmission terms in host-microparasite models: numbers, densities and areas." Epidemiology and Infection 129 (1) : 147153.

[3] Fenton, A., Fairbairn, J. P., Norman, R., and Hudson, P. J. 2002. "Parasite transmission: reconciling theory and reality." Journal of Animal Ecology 71 (5): 893905.

[4] Jeger, M. J., van den Bosch, F., Madden, L. V., and Holt, J. 1998. "A model for analysing plant-virus transmission characteristics and epidemic development." Math. Med. Biol., 15 (1): 118.

[5] Madden, L. V., M. J. Jeger, and F. van den Bosch. 2000. A Theoretical Assessment of the Effects of Vector-Virus Transmission Mechanism on Plant Virus Disease Epidemics. Phytopathology 90 (6): 57694.

[6] McCallum, H., Barlow, N., and Hone, J. (2001). "How should pathogen transmission be modelled?" Trends in Ecology and Evolution, 16 (6): 295300.

[7] Rudolf, V. H. W. and Antonovics, J. 2005. "Species coexistence and pathogens with frequency-dependent transmission." American Naturalist 166 (1): 112118.
