# Supplementary figures and images for: A method of determining where to target surveillance efforts in heterogeneous epidemiological systems
Source: PLoS Comput Biol. 2017 Aug 28;13(8):e1005712. doi: 10.1371/journal.pcbi.1005712 (PMC5591013; doi:10.1371/journal.pcbi.1005712)

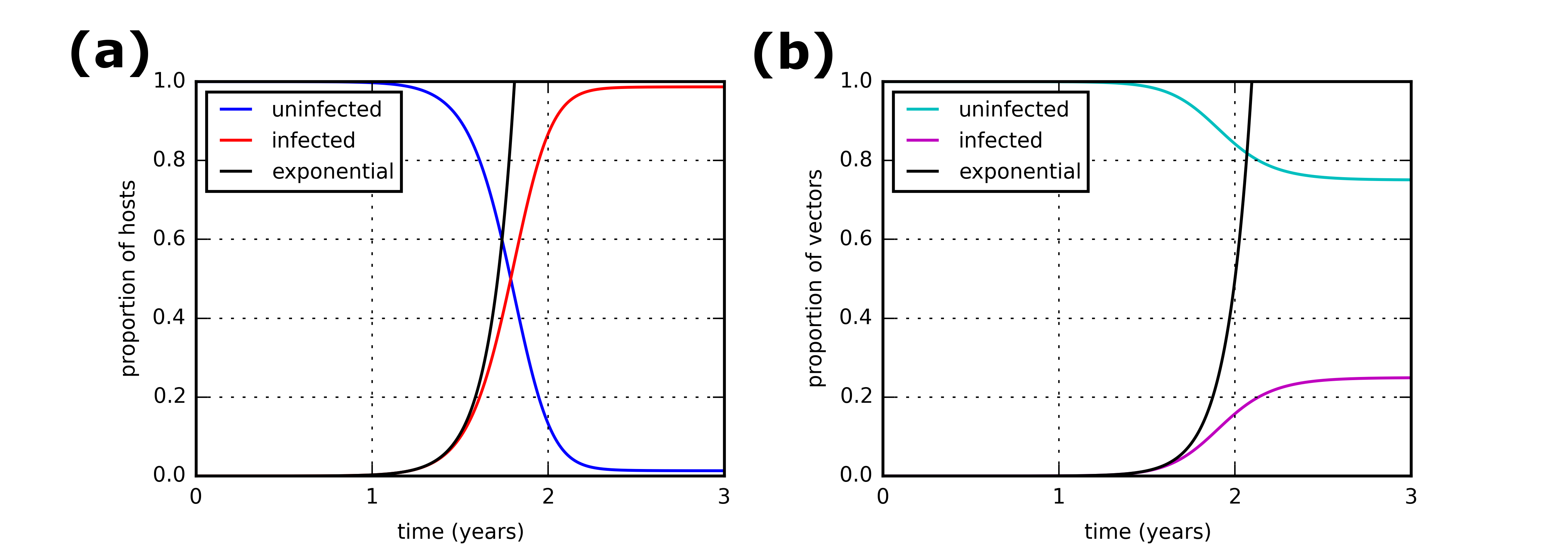

Supplement: S1 Fig — Host and vector transmission dynamics in HLB model over the course of two years. Hosts are shown in panel (a) and vectors in panel (b). The relative densities of hosts and vectors for both models was fixed in order to give an R0 estimate of 100. (TIF) [file pcbi.1005712.s007.tif]

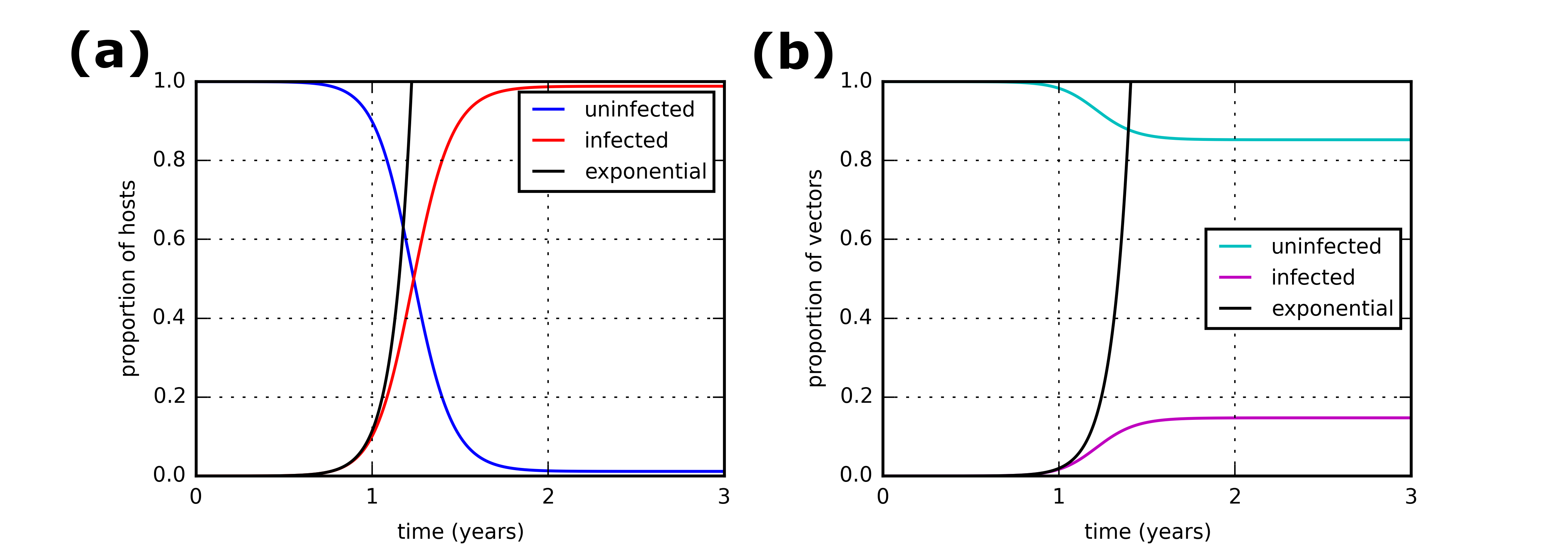

Supplement: S2 Fig — Host and vector transmission dynamics in the tristeza model over the course of two years. Hosts are shown in panel (a) and vectors in panel (b). The relative densities of hosts and vectors for both models was fixed in order to give an R0 estimate of 100. (TIF) [file pcbi.1005712.s008.tif]

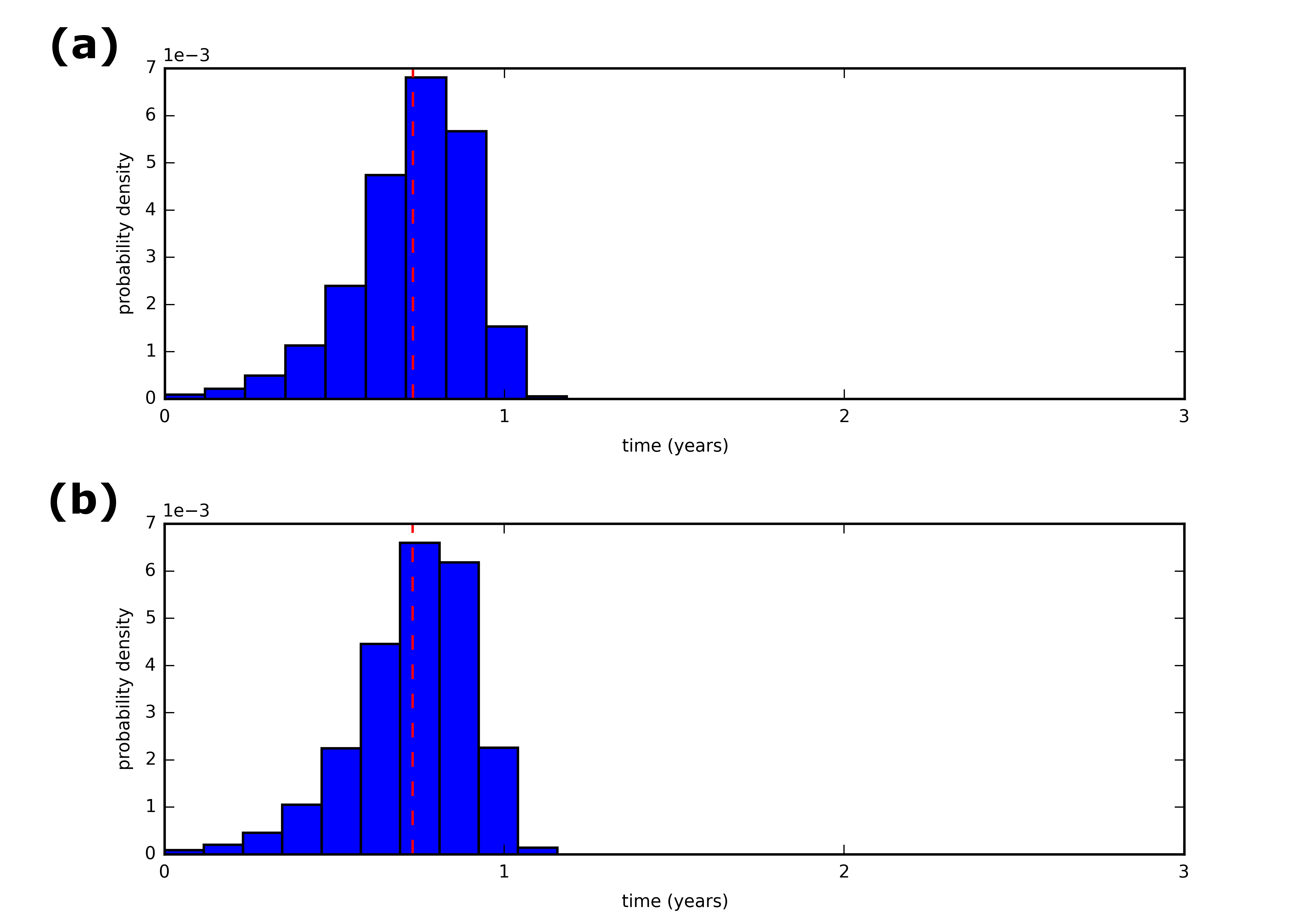

Supplement: S3 Fig — Simulated distribution of time of first detection at the cost ratio threshold with a sampling ‘cost’ equivalent to that of 800 hosts every 28 days in the HLB model (i.e. either 800 hosts or 6,382 vectors). Panel (a) shows the results predicted when sampling 800 hosts and no vectors, and Panel (b) shows those predicted when sampling 6,382 vectors and no hosts. The dotted lines show the average time at first detection. (TIF) [file pcbi.1005712.s009.tif]

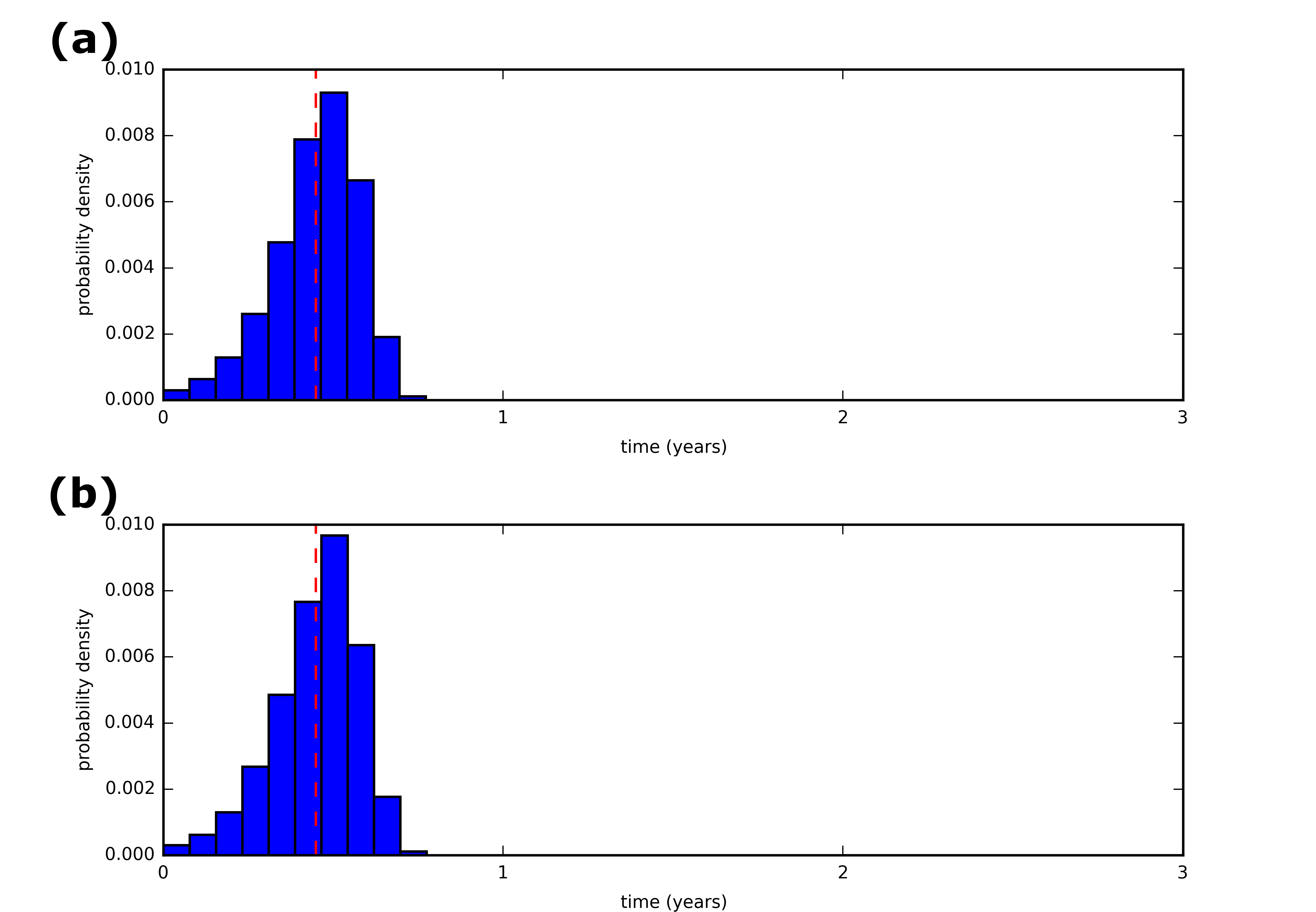

Supplement: S4 Fig — Predicted distribution of time of first detection at the cost ratio threshold with a sampling ‘cost’ equivalent to that of 800 hosts every 28 days in the tristeza model (i.e. either 800 hosts or 4,687 vectors). Panel (a) shows the results predicted when sampling 800 hosts and no vectors, and Panel (b) shows those predicted when sampling 4,687 vectors and no hosts. The dotted lines show the average time at first detection. (TIF) [file pcbi.1005712.s010.tif]

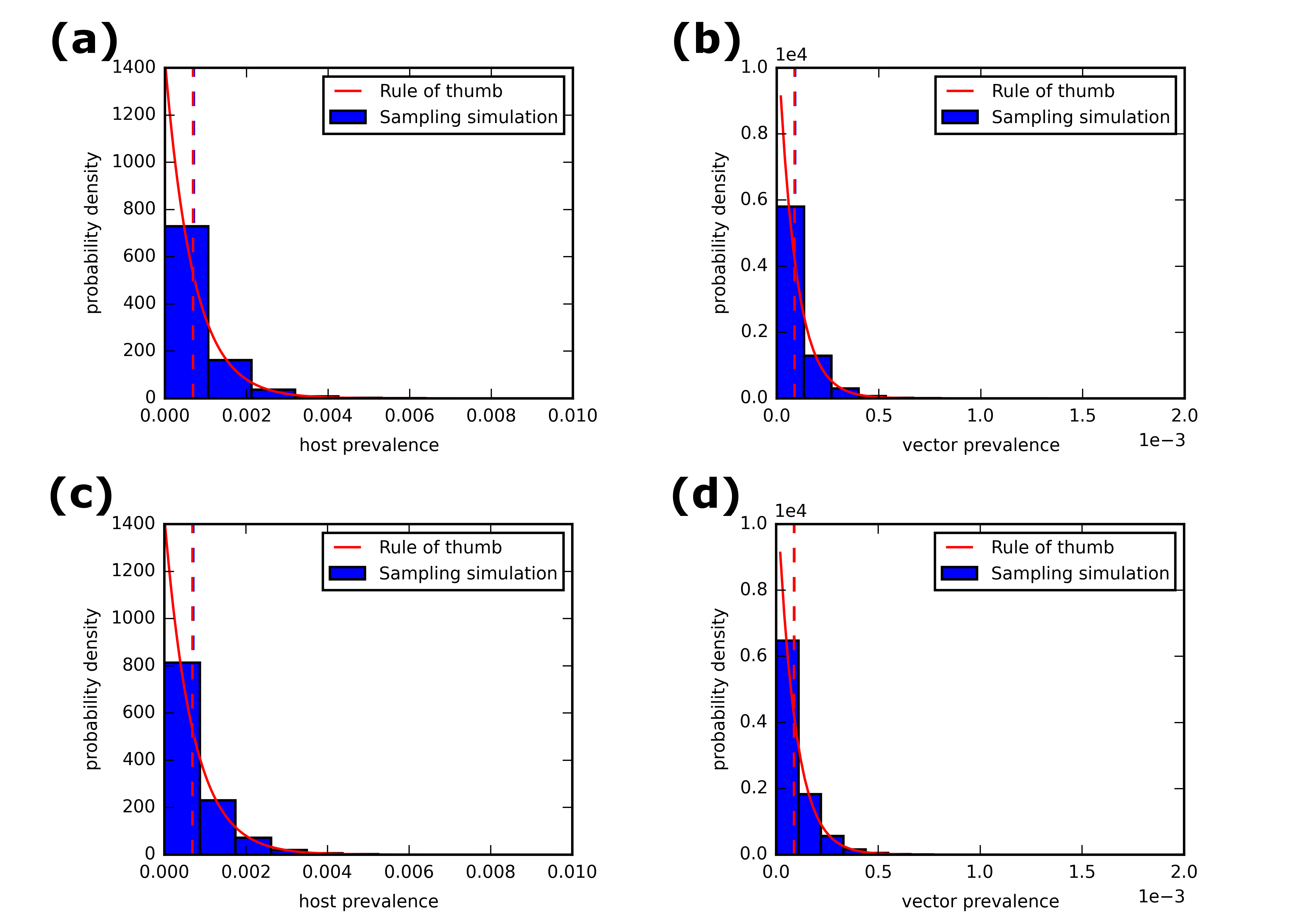

Supplement: S5 Fig — Predicted distribution of prevalence at first detection at the cost ratio threshold with a sampling ‘cost’ equivalent to that of 800 hosts every 28 days in the HLB model (i.e. either 800 hosts or 6,382 vectors), using both model simulation and the heuristic (‘rule of thumb’). Host prevalence at first detection is shown in panels (a) and (c), and vector prevalence in panels (b) and (d). Panels (a) and (b) show the results when sampling only from hosts, and panels (c) and (d) show those predicted when vectors alone are sampled. Dotted lines show the mean prevalence at first detection. (TIF) [file pcbi.1005712.s011.tif]

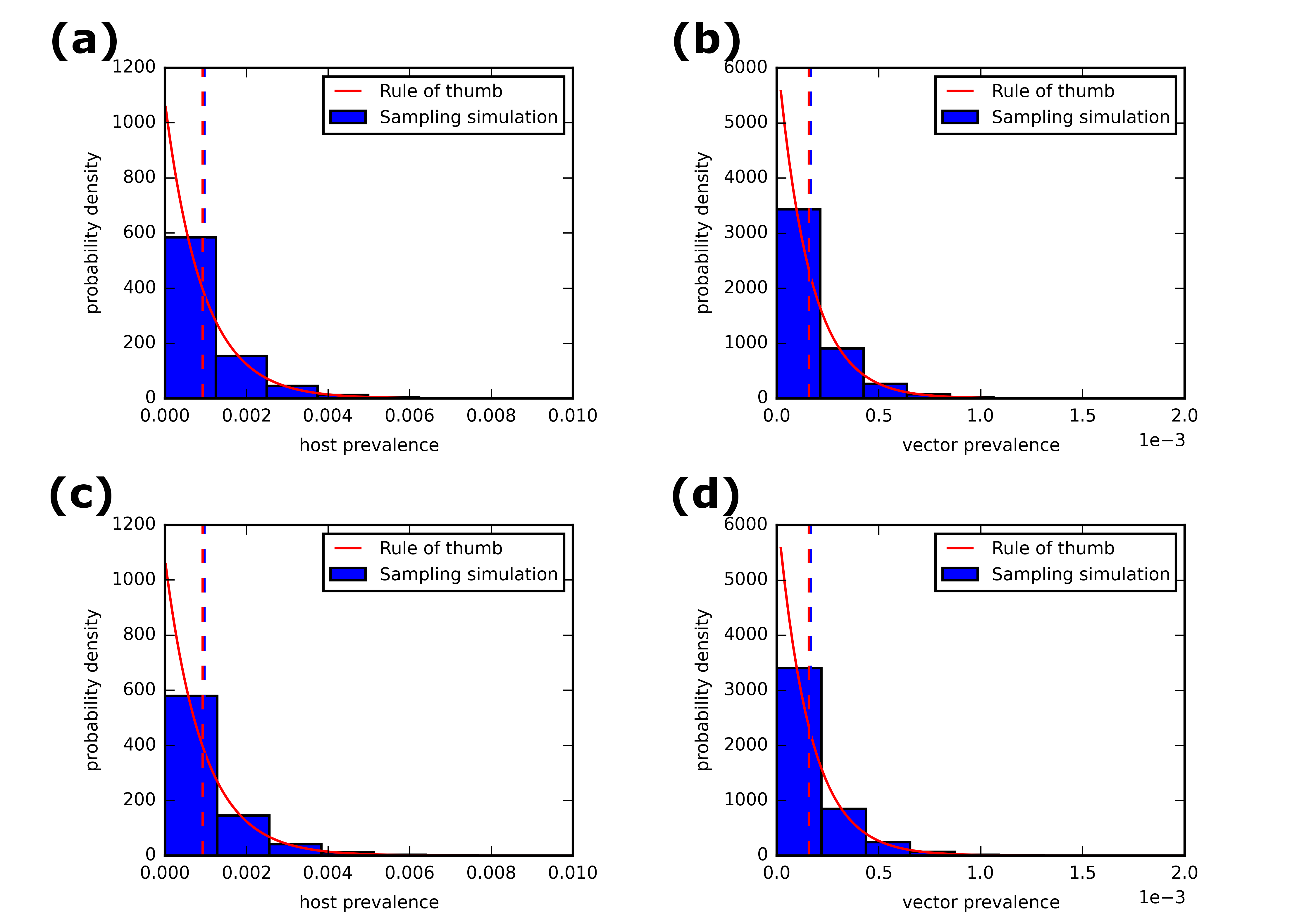

Supplement: S6 Fig — Predicted distribution of prevalence at first detection at the cost ratio threshold with a sampling ‘cost’ equivalent to that of 800 hosts every 28 days in the tristeza model (i.e. either 800 hosts or 4,687 vectors), using both model simulation and the heuristic (‘rule of thumb’). Host prevalence at first detection is shown in panels (a) and (c), and vector prevalence in panels (b) and (d). Panels (a) and (b) show the results when sampling only from hosts, and panels (c) and (d) show those predicted when vectors alone are sampled. Dotted lines show the mean prevalence at first detection. (TIF) [file pcbi.1005712.s012.tif]

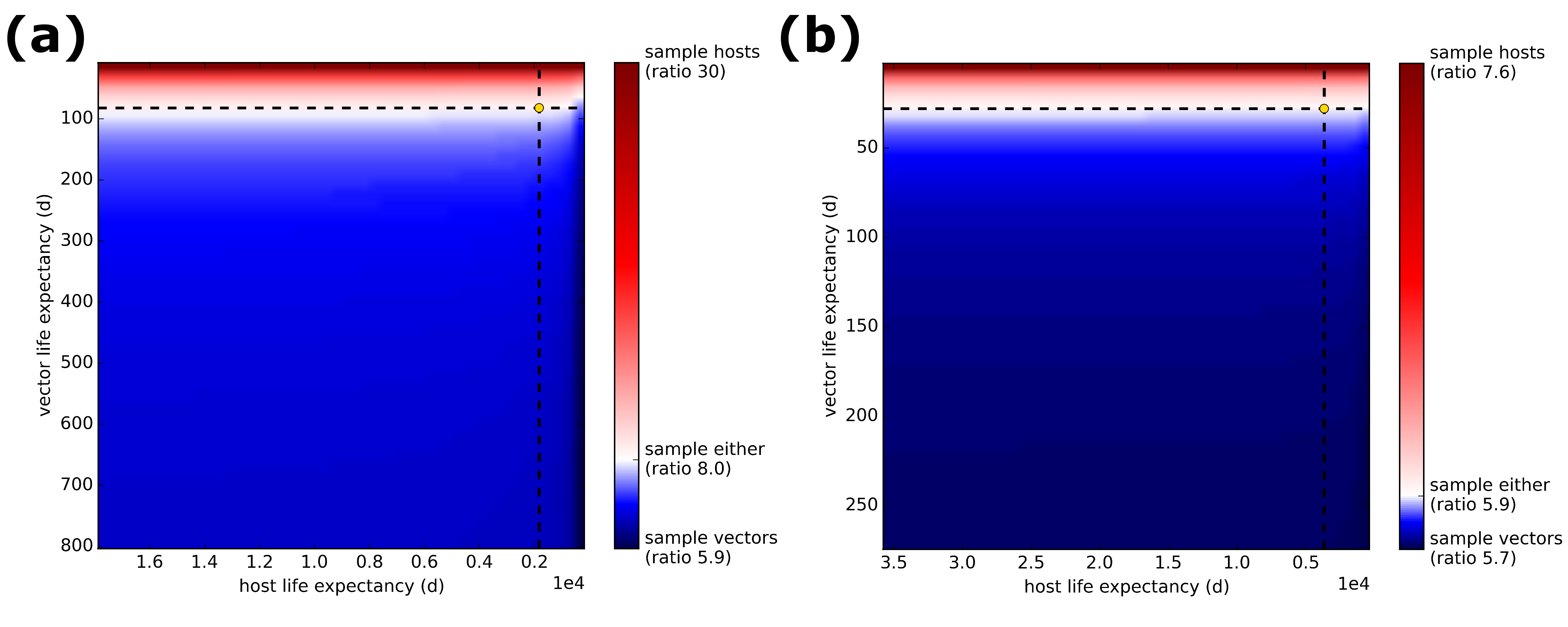

Supplement: S7 Fig — Effect of varying longevity parameters (μ) on the suggested group of sampling for the HLB model (panel (a)) and the tristeza model (panel (b)), assuming a sampling cost ratio at the threshold (8 for HLB, 6 for Tristeza). The intersection of the dashed lines shows the current parameter values. The colour gradient relates to the ratio [(νhρh)(νvρv)], and is shown on the log scale. Red indicates a ratio greater than the cost ratio (suggesting host sampling) and blue indicates a ratio less than the cost ratio (suggesting vector sampling). (TIF) [file pcbi.1005712.s013.tif]

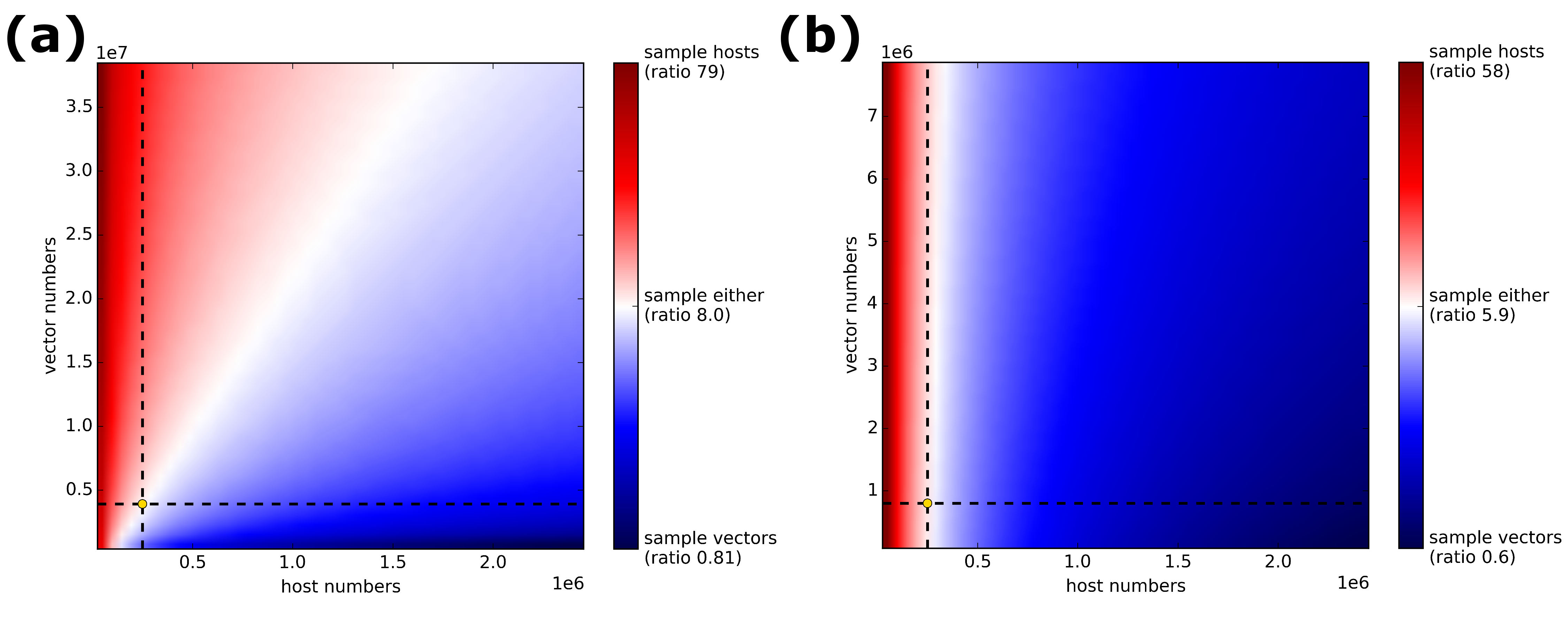

Supplement: S8 Fig — Effect of varying numbers of hosts and vectors (ρ parameters) on the suggested stratum of sampling for the HLB model (panel (a)) and the tristeza model (panel (b)), assuming a sampling cost ratio at the threshold (8 for HLB, 6 for Tristeza). The intersection of the dashed lines shows the current parameter values. The colour gradient relates to the ratio [(νhρh)(νvρv)], and is shown on the log scale. Red indicates a ratio greater than the cost ratio (suggesting host sampling) and blue indicates a ratio less than the cost ratio (suggesting vector sampling). (TIF) [file pcbi.1005712.s014.tif]

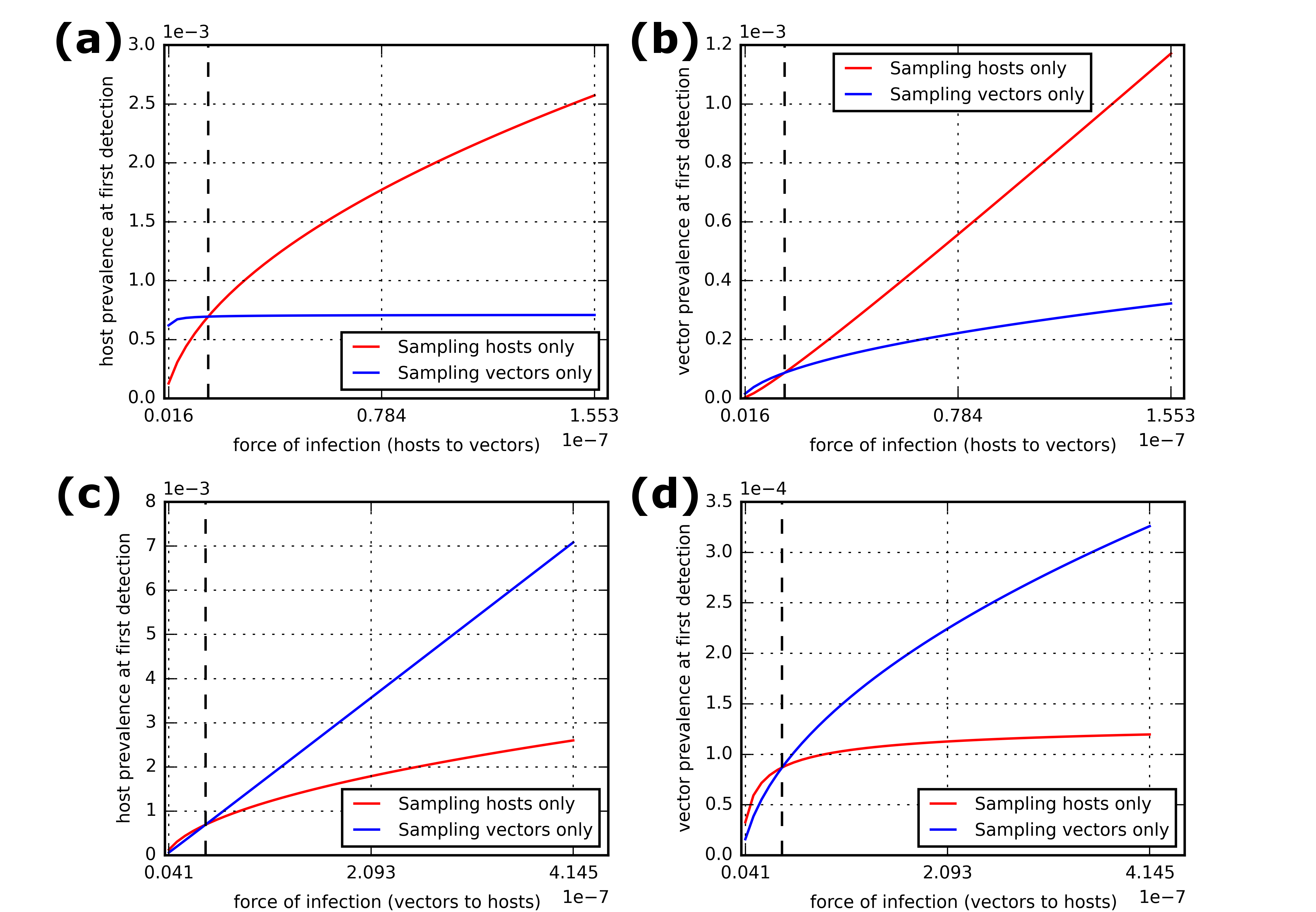

Supplement: S9 Fig — Effect of varying transmission rates (β parameters) on the mean prevalence at first detection for the HLB model (host prevalence shown in panels (a) and (c) and vector prevalence in panels (b) and (d)). Red lines show the estimated prevalence when 800 hosts are sampled every 28 days, and blue lines show the estimated prevalence when 6,382 vectors are sampled every 28 days. Plots in panels (a) and (b) show the effect of varying host to vector transmission, and those in panels (c) and (d) show the effect of varying vector to host transmission. The dashed line shows the parameter value used in the model. The transmission parameters have units of ‘infections per host per vector per day’ (TIF) [file pcbi.1005712.s015.tif]

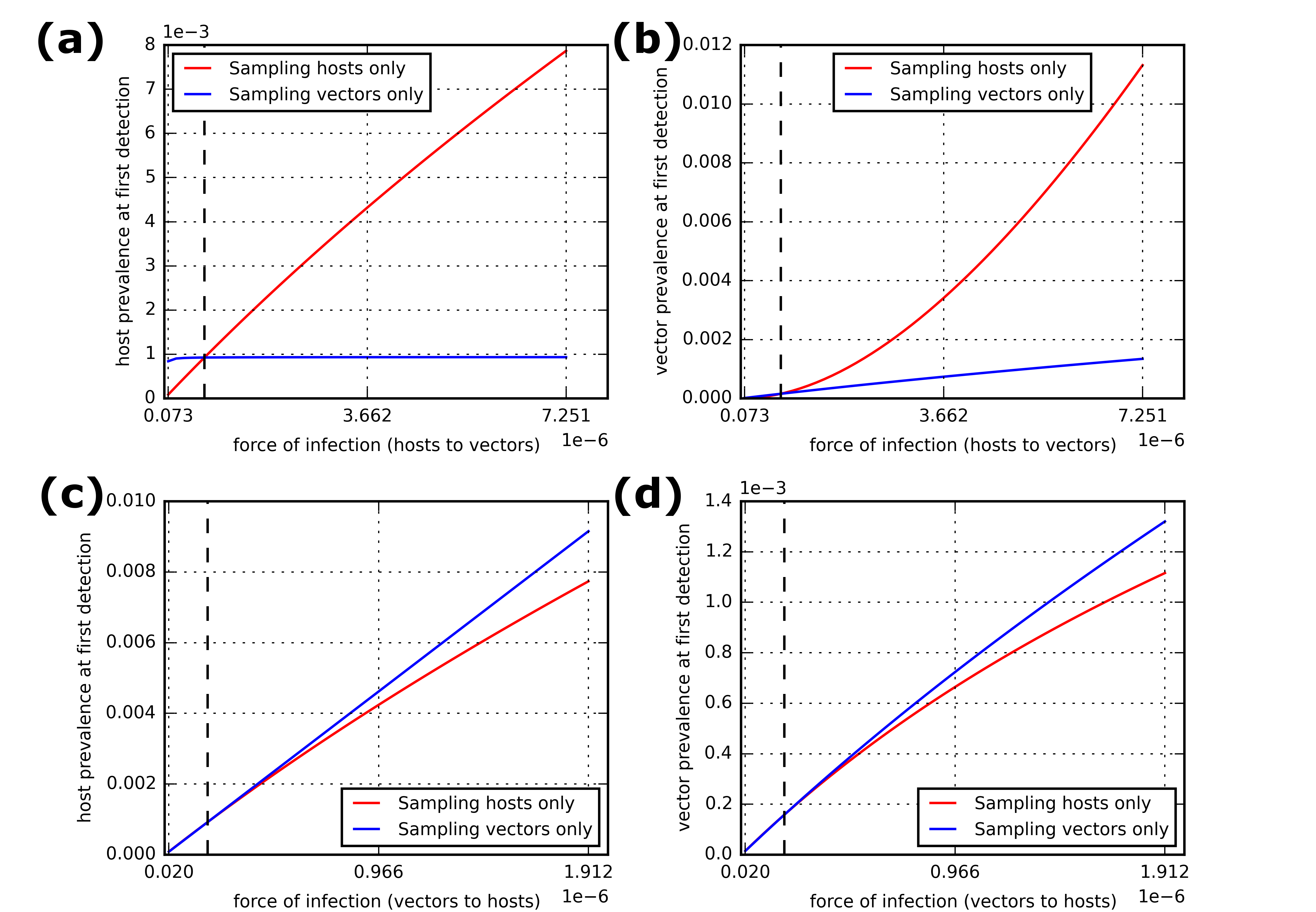

Supplement: S10 Fig — Effect of varying transmission rates (β parameters) on the mean prevalence at first detection for the tristeza model (host prevalence shown on the left and vector prevalence on the right). Red lines show the estimated prevalence when 800 hosts are sampled every 28 days, and blue lines show the estimated prevalence when 4,687 vectors are sampled every 28 days. Plots in panels (a) and (b) show the effect of varying host to vector transmission, and those in panels (c) and (d) show the effect of varying vector to host transmission. The dashed line shows the parameter value used in the model. The transmission parameters have units of ‘infections per host per vector per day’ (TIF) [file pcbi.1005712.s016.tif]

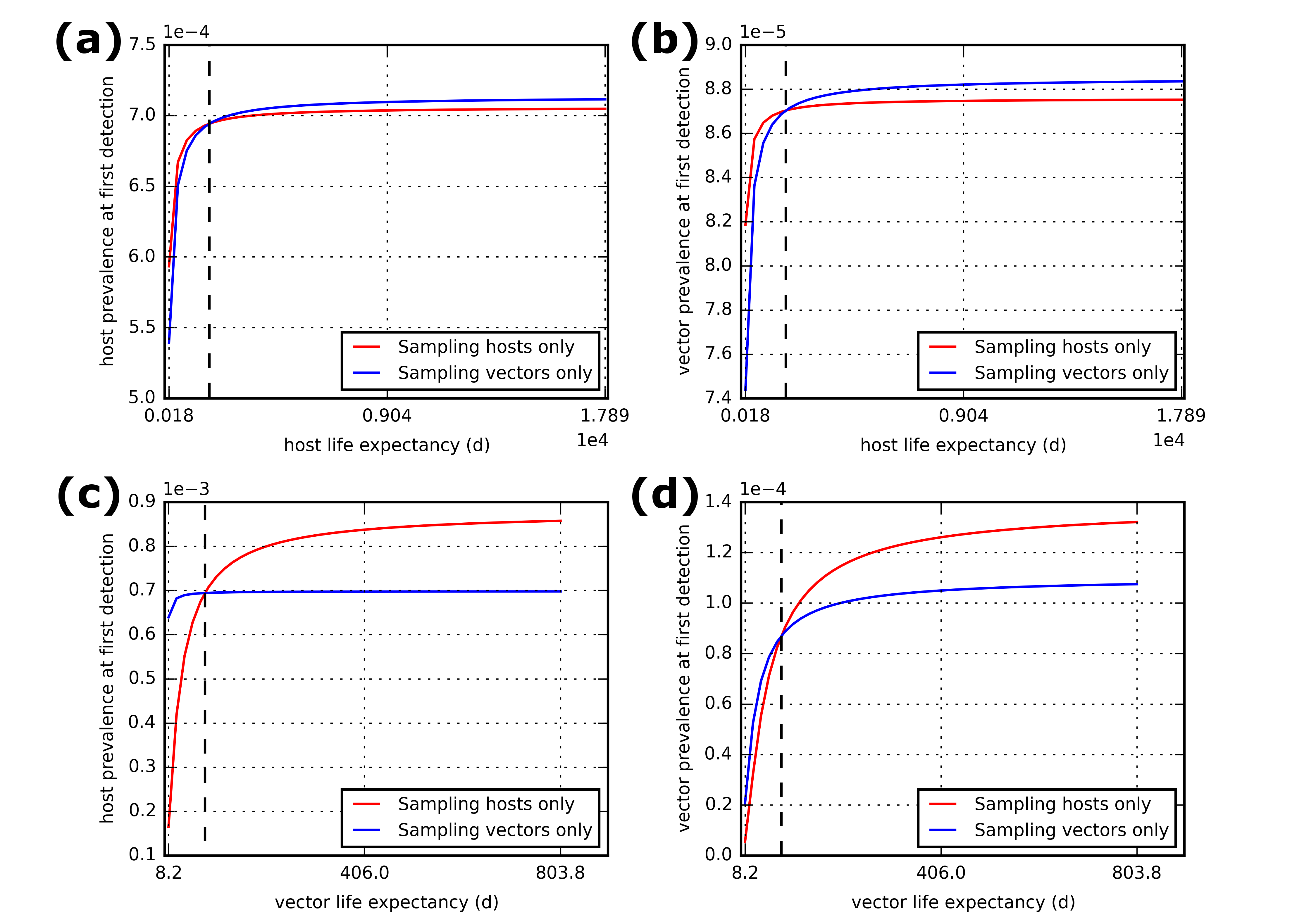

Supplement: S11 Fig — Effect of varying longevity (μ parameters) on the mean prevalence at first detection for the HLB model (host prevalence shown on the left and vector prevalence on the right). Red lines show the estimated prevalence when 800 hosts are sampled every 28 days, and blue lines show the estimated prevalence when 6,382 vectors are sampled every 28 days. Plots in panels (a) and (b) show the effect of varying host longevity, and those in panels (c) and (d) show the effect of varying vector longevity. The dashed line shows the parameter value used in the model. (TIF) [file pcbi.1005712.s017.tif]

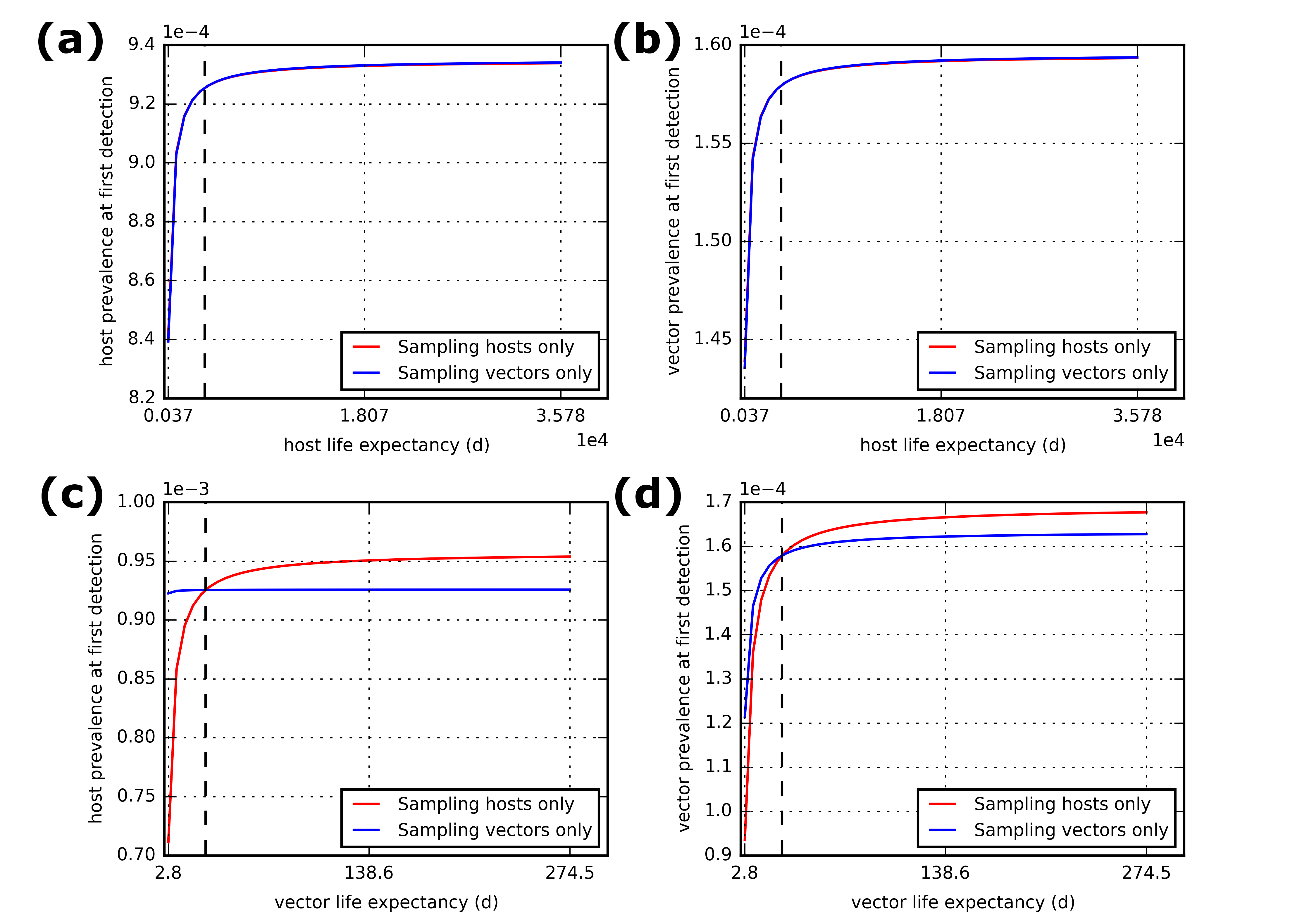

Supplement: S12 Fig — Effect of varying longevity (μ parameters) on the mean prevalence at first detection for the tristeza model (host prevalence shown on the left and vector prevalence on the right). Red lines show the estimated prevalence when 800 hosts are sampled every 28 days, and blue lines show the estimated prevalence when 4,687 vectors are sampled every 28 days. Plots in panels (a) and (b) show the effect of varying host longevity, and those in panels (c) and (d) show the effect of varying vector longevity. The dashed line shows the parameter value used in the model. (TIF) [file pcbi.1005712.s018.tif]
